# Epidemiological features and trends in the mortality rates of 10 notifiable respiratory infectious diseases in China from 2004 to 2020: Based on national surveillance

**DOI:** 10.3389/fpubh.2023.1102747

**Published:** 2023-02-17

**Authors:** Na Zhao, Supen Wang, Lan Wang, Yingying Shi, Yixin Jiang, Tzu-Jung Tseng, Shelan Liu, Ta-Chien Chan, Zhiruo Zhang

**Affiliations:** ^1^School of Ecology and Environment, Anhui Normal University, Wuhu, Anhui, China; ^2^Collaborative Innovation Center of Recovery and Reconstruction of Degraded Ecosystem in Wanjiang Basin Co-founded by Anhui Province and Ministry of Education, Anhui Normal University, Wuhu, China; ^3^College of Life Sciences, Anhui Normal University, Wuhu, Anhui, China; ^4^Department of Geriatrics, The First Affiliated Hospital, Zhejiang University School of Medicine, Zhejiang, China; ^5^Research Center for Humanities and Social Sciences, Academia Sinica, Taipei, Taiwan; ^6^Department of Infectious Diseases, Zhejiang Provincial Centre for Disease Control and Prevention, Hangzhou, Zhejiang, China; ^7^School of Public Health, Lanzhou University, Lanzhou, Gansu, China; ^8^School of Public Health, Shanghai Jiao Tong University School of Medicine, Shanghai, China

**Keywords:** respiratory infectious diseases, deaths, case fatality rate, mortality, epidemiological features and trends

## Abstract

**Objectives:**

The aim of this study is to describe, visualize, and compare the trends and epidemiological features of the mortality rates of 10 notifiable respiratory infectious diseases in China from 2004 to 2020.

**Setting:**

Data were obtained from the database of the National Infectious Disease Surveillance System (NIDSS) and reports released by the National and local Health Commissions from 2004 to 2020. Spearman correlations and Joinpoint regression models were used to quantify the temporal trends of RIDs by calculating annual percentage changes (APCs) in the rates of mortality.

**Results:**

The overall mortality rate of RIDs was stable across China from 2004 to 2020 (*R* = −0.38, *P* = 0.13), with an APC per year of −2.2% (95% CI: −4.6 to 0.3; *P* = 0.1000). However, the overall mortality rate of 10 RIDs in 2020 decreased by 31.80% (*P* = 0.006) compared to the previous 5 years before the COVID-19 pandemic. The highest mortality occurred in northwestern, western, and northern China. Tuberculosis was the leading cause of RID mortality, and mortality from tuberculosis was relatively stable throughout the 17 years (R = −0.36, *P* = 0.16), with an APC of −1.9% (95% CI −4.1 to 0.4, *P* = 0.1000). Seasonal influenza was the only disease for which mortality significantly increased (*R* = 0.73, *P* = 0.00089), with an APC of 29.70% (95% CI 16.60–44.40%; *P* = 0.0000). The highest yearly case fatality ratios (CFR) belong to avian influenza A H5N1 [687.5 per 1,000 (33/48)] and epidemic cerebrospinal meningitis [90.5748 per 1,000 (1,010/11,151)]. The age-specific CFR of 10 RIDs was highest among people over 85 years old [13.6551 per 1,000 (2,353/172,316)] and was lowest among children younger than 10 years, particularly in 5-year-old children [0.0552 per 1,000 (58/1,051,178)].

**Conclusions:**

The mortality rates of 10 RIDs were relatively stable from 2004 to 2020 with significant differences among Chinese provinces and age groups. There was an increased mortality trend for seasonal influenza and concerted efforts are needed to reduce the mortality rate of seasonal influenza in the future.

## Introduction

The Global Burden of Disease Study 2017 (GBD 2017) analysis indicates that the major factors affecting disability-adjusted life years (DALYs) globally are communicable diseases, including lower respiratory infections, malaria, diarrhea diseases, HIV/acquired immunodeficiency syndrome (AIDS), and tuberculosis (1). For developed and developing countries, respiratory infectious diseases (RIDs) impose a large health burden on health systems and consistently rank among the most fatal diseases (2–4). The severe acute respiratory syndrome coronavirus-2 (SARS-CoV-2) pandemic, which began in 2020, indicated that RIDs are still a global critical health threat.

As the country with the largest population in the world, China faces great pressure in the prevention and control of RIDs, which are still severe health and financial burdens due to their relatively high incidence rates (5–7). Infectious disease mortality in China has been reported previously; surveys have covered the period of 1986–2016 (39 notifiable infectious diseases) (8), 2004–2013 year (45 notifiable infectious diseases) (9), 2010–2019 (44 notifiable infectious diseases) (10), and 1990–2019 (the burden of upper respiratory infections without disease types) (11), and the epidemic pattern of RIDs has changed significantly in recent years. For example, China is experiencing an unprecedented rise in scarlet fever, pertussis, and multi-drug-resistant tuberculosis (12–14). There are several factors that contribute to these changes: First, shifts in the emerging respiratory infectious pathogens or subtype shifts have been recurring every 2–3 years (5, 15–21). Second, changes in demographic aspects and structure due to urbanization, population growth, aging, birth rates, etc. will change the number of people susceptible to RIDs. Third, climate change and environmental factors may affect the risk of RID outbreaks (9, 22, 23). To account for these changes, it is necessary to periodically assess the epidemiological characteristics of the mortality rates of RIDs on a national and provincial level.

This study aims to use national notifiable infectious diseases surveillance data to uncover the latest and overall picture of the epidemiological features and trends in the age- or province-specific mortality rates of 10 notifiable RIDs in China from 2004 to 2020. Based on literature searches in PubMed, our study reports the yearly mortality rates, case fatality rates, spatiotemporal and age distribution, and their trends of 10 notifiable RIDs from 2004 to 2020 in mainland China, as well as the impact of the continuous containment and mitigation strategies for COVID-19 on the mortality rates of the other 10 notifiable RIDs. Understanding trends in respiratory infectious deaths and changes in the leading causes of disease burden over time is needed for decision-makers to better set policies and priorities for action.

## Methods

### Data sources

Data were obtained from the database of the National Infectious Disease Surveillance System (NIDSS) and official reports on national notifiable infectious diseases released by the National and local Health Commissions. Using the NIDSS system, hospitals and clinics from all 31 provinces in mainland China can directly report, in real time, clinically diagnosed cases, suspected cases, or confirmed cases of infectious diseases. A total of 40 notifiable infectious diseases are divided into three classes (A, B, and C). Epidemic reports are time-sensitive; all class A infectious disease cases and class B pulmonary anthrax and SARS cases should be reported through the network within 2 h of diagnosis, whereas the remaining class B and C infectious diseases should be reported within 24 h (8, 9, 24). The outbreak of severe acute respiratory syndrome (SARS) in 2003 led to the establishment of a web-based, timely reporting system (NIDSS) in China in 2004, which has improved the efficiency of the infectious disease surveillance system. The strategies based on non-pharmaceutical interventions that were used to contain the COVID-19 pandemic in China appear to be effective for other infectious diseases. Therefore, this study uses national surveillance data from 2004 to 2020. The annual population data for the years 2004–2020 were collected from the data published on the website of the Chinese National Bureau of Statistics at the end of each year.

### Case diagnosis criteria

NIDSS data includes clinically diagnosed cases, suspected cases, and confirmed cases. The case definitions and diagnostic criteria are approved and issued by the China National Standardization Administration Committee. Details of case definitions and diagnostic criteria for all 10 respiratory diseases are available in Supplementary Tables 1–11.

### Data collection

According to the transmission mode, clinical severity, and epidemic intensity, this study obtained mortality data for 10 respiratory diseases from 40 notifiable infectious diseases (except for COVID-19) in mainland China, where these respiratory infections are the major contributors to the burden of infectious disease, affect all age groups, and have been identified in all provinces. Among them, six belong to Class B (TB, epidemic cerebrospinal meningitis, H5N1 avian influenza, measles, pertussis, and scarlet fever) and four belong to Class C [seasonal influenza, 2009 pandemic influenza A(H1N1) (pH1N1), mumps, and rubella]. Data on all respiratory diseases cover the period of 2004–2020, except pH1N1 (which covered 2009–2013), which was grouped with seasonal influenza after 2013. We collected data on the number of cases and deaths, incidence rates, and mortality rates, stratified by age groups and provinces.

### Data analyses

This study includes 50,595 deaths from Jan 1, 2004 to Dec 31, 2020. We used an R package (version 4.2.0), Joinpoint Regression Analysis software (version 4.5.0.1; the National Cancer Institute, details in Supplementary material), ArcGIS 10.2 (ArcMap, version 10.3; ESRI Inc., Redlands, CA, USA), and SaTScan.9.6 (http://www.satscan.org/) to analyze trends, annual percentage changes (APC), and the spatiotemporal distribution of the mortality rate (4, 9, 13, 25).

### Statistical analysis

The case fatality ratio (per 1,000) is the number of annual deaths divided by the number of annual incident cases. Statistical analysis was performed using ggplot2 (version 3.3.6) and ggpubr (version 0.4.0) packages. We used the *x*^2^ test to compare the different proportions of infected people by mortality year and age. For all analyses, probabilities were two-tailed, spearman correlations were calculated, and a *P*-value of <0.05 was considered statistically significant. We express trends as annual percentage changes (Supplementary material). We used the *Z*-test to assess whether an annual percentage change is significantly different from zero. In describing trends, we use the terms “increase” and “decrease” when the slope (annual percentage change) is significant (*P* < 0.05). We use the term “stable” to refer to a non-significant annual percentage change (*P* ≥ 0.05) and indicate that the incidence is maintained at a perennially stable level or that the incidence is perennially unreported or only reported sporadically.

### Patient and public involvement

There was no patient or public involvement in this research.

## Results

### Overall trends of mortality and number of deaths caused by 10 RIDs from 2004 to 2020

From 2004 to 2020, a total of 50,595 deaths caused by 10 RIDs were recorded in China, accounting for 18.39% (50,595/275,071) of deaths caused by 39 notifiable infectious diseases in China. The yearly average deaths totaled 2,976 (95% CI 2,606–3,346) (Table 1).

**Table 1:** The mortality data for 10 notifiable respiratory infectious diseases in China from 2004 to 2020.

**Disease name**	**No. of deaths**	**Percentage in overall respiratory deaths (%)**	**Percentage in all infectious deaths (%)**	**No. of annual deaths (95%CI)**	**CFR (per 1,000)**
All 10 RIDs	50,595	100	18.39	2,976 (2,606–3,346)	1.6411
TB	47,374	93.63	17.22	2,787 (2,472–3,101)	2.9131
Epidemic cerebrospinal meningitis	1,010	2.00	0.37	59 (25–94)	90.5748
Seasonal influenza	711	1.41	0.26	42 (6–78)	0.0956
pH1N1	916	1.18	0.33	183 (−149 to 515)	5.2125
Measles	476	0.94	0.17	28 (14–42)	0.5938
Avian influenza H5N1	33	0.07	0.01	2.1 (0.9–3.3)	687.5000
Pertussis	33	0.07	0.01	1.9 (0.9–3.0)	0.3034
Mumps	25	0.05	0.01	1.5 (0.6–2.3)	0.0053
Scarlet fever	11	0.02	0.004	0.6 (0.2–1.1)	0.0146
Rubella	6	0.01	0.00	0.4 (0.1–0.6)	0.0103

CFR, case fatality rate; TB, tuberculosis; pH1N1, 2009 pandemic influenza H1N1 virus.

**Figure 1:**
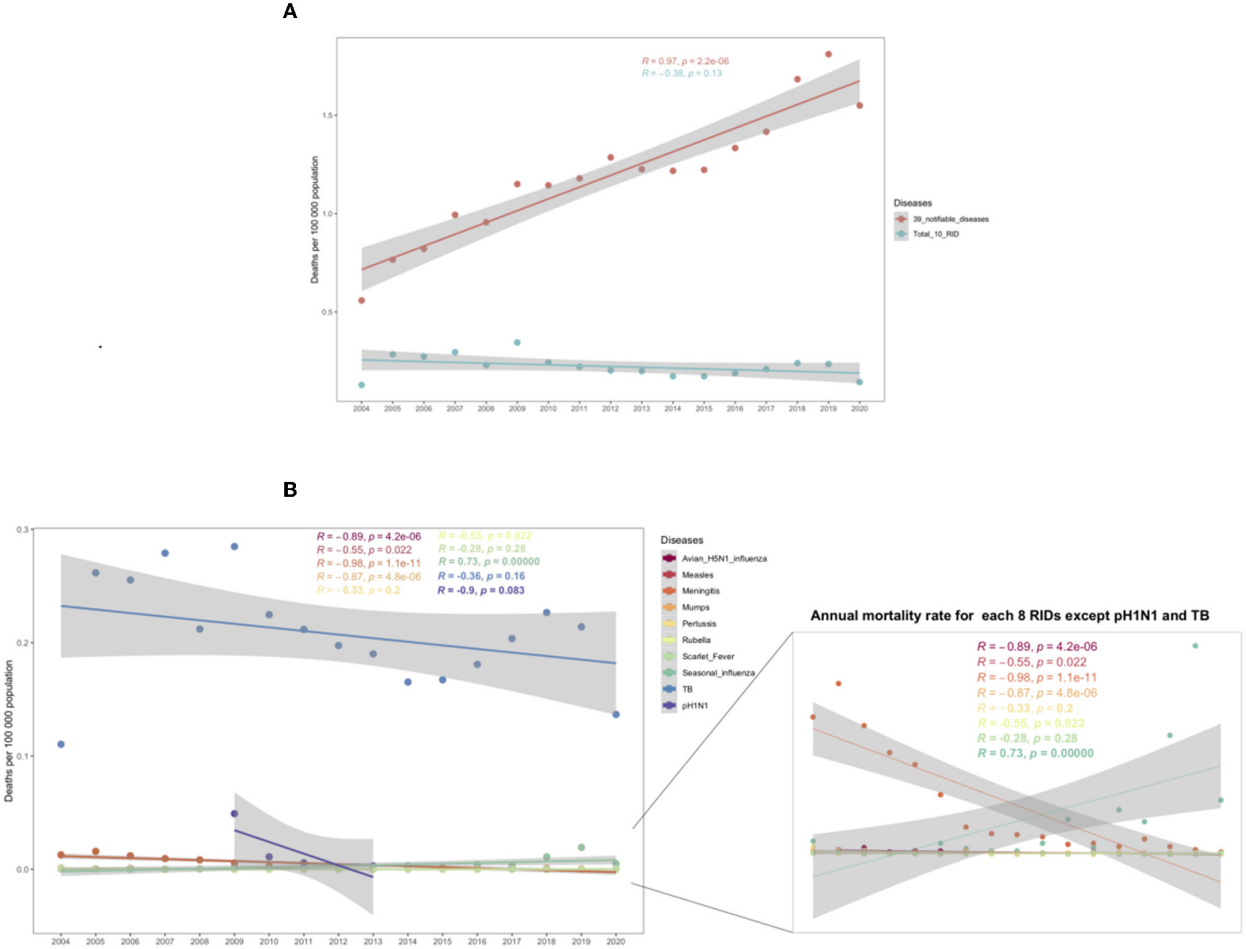
Trends in annual mortality rates from 2004 to 2020. **(A)** Annual mortality for 39 notifiable diseases and all 10 respiratory infectious diseases (RIDs). **(B)** Annual mortality for each RID. The *P*-value indicates the coefficient of the spearman correlation. The shadings indicate the 95% confidence intervals (CI) and the colors indicate different diseases.

Regarding the mortality rates of the 39 notifiable infectious diseases, a significantly increasing trend was found from 2004 to 2020 (*R* = 0.97, *P* = 2.2e-06, Figure 1), including a rapid increase in the yearly mortality from 2004 to 2019 and a reduction in 2020.

In terms of the 10 RIDs, the mortality rate showed a stable trend in the 17 years from 2004 to 2020 (*R* = −0.38, *P* = 0.13, Figure 1), although the mortality rate of all RIDs in 2020 remained higher than that in 2004 (0.1422 vs. 0.1274 per 100,000: an increase of 20.47%, Figure 1; Table 2). Meanwhile, the Joinpoint regression indicates that the APC of age-adjusted mortality was −2.2% (95% CI −4.6 to 0.3%; *P* = 0.1000) across the whole of China from 2004 to 2020 (Table 3).

**Table 2:** Changes in number of cases, number of deaths, and mortality (per 100,000) for 10 respiratory infectious diseases in China, 2004 vs. 2020.

**Diseases**	**2004**		**2020**		**Overall trends (2020 vs. 2004)**	**Changed areas % (** ***n*** **)**	**Areas with the greatest changes**
	**No. of cases (no. of deaths)**	**Mortality**	**No. of cases (no. of deaths)**	**Mortality**			
All 10 RIDs	1,367 500 (1,656)	0.1274	1,969 082 (1,995)	0.1422	+20.47%	48.39% (15/31) increased	Xinjiang, Liaoning, Hainan, Chongqing, Ningxia
TB	970,279 (1,435)	0.1104	670,538 (1,919)	0.1368	+33.73%	54.84% (17/31) increased	Xinjiang, Liaoning, Hainan, Chongqing, Ningxia
Epidemic cerebrospinal meningitis	2,698 (165)	0.0127	50 (3)	0.0002	−98.18%	87.10% (27/31) decreased	Beijing, Tibet, Qinghai, Guizhou
Seasonal influenza	49,496 (15)	0.0012	1,145,278 (70)	0.0050	+366.67%	90.32% (28/31) increased	Beijing, Chongqing, Shandong
pH1N1^*^	147,337 (10,841)^*^	0.0491^*^	652 (39)^*^	0.0029^*^	−94.09%^*^	93.55% (29/31) decreased^*^	Beijing, Qinghai, Hebei, Ningxia^*^
Measles	70,549 (26)	0.0020	856 (0)	0	−100%	32.25% (10/31) decreased	Guizhou, Xinjiang
Avian influenza H5N1	(—)	(—)	0 (0)	0	(—)	(—)	(—)
Pertussis	4,705 (9)	0.0007	4,475 (1)	0.0001	−85.71%	12.90% (4/31) decreased	Qinghai, Xinjiang, Chongqing, Sichuan
Mumps	226,819 (4)	0.0003	129,120 (1)	0.0001	−66.67%	6.45% (2/31) decreased	Shannxi, Guangxi
Scarlet Fever	18,939 (1)	0.0001	16,564 (1)	0.0001	0	3.23% (1/31) reported	Guangdong
Rubella	24,015 (1)	0.0001	2,201 (0)	0	−100%	3.23% (1/31) decreased	Henan

Overall trends = (mortality in 2020 – mortality in 2004)/mortality in 2004 × 100%.

* pH1N1, the full period from 2009 to 2013.

Overall trend for pH1N1 = (mortality in 2013 – mortality in 2009)/mortality in 2009 × 100%.

“+” means “increased” and “–” means “decreased”.

(—), no cases or numbers reported in the system for each infectious disease.

**Table 3:** Annual percentage change in incidence rate of all 10 RIDs, from 2004 to 2020.

**Diseases**	**Trends**	**Annual percentage change (95% CI)**	***P*-value^†^**
All 10 RIDs	Stable	−2.2 (−4.6 to 0.3)	0.1
TB	Stable	−1.9 (−4.1 to 0.4)	0.1
Epidemic cerebrospinal meningitis	Decrease	−21.8 (−24.9 to −18.5)	< 0.05
Seasonal influenza	Increase	29.7 (16.6–44.4)	< 0.05
pH1N1^*^	Decrease	−59.4 (−78.1 to −24.8)	< 0.05
Measles	Decrease	−8.3 (−14.6 to −1.6)	< 0.05
Avian influenza H5N1^‡^	Decrease	−13.5 (−20 to 6.6)	< 0.05
Pertussis	Stable	−7.9 (−17.2 to 2.3)	0.1
Mumps	Decrease	−11.3 (−16.4 to −5.9)	< 0.05
Scarlet fever	Stable	1.4 (−1.9 to 4.8)	0.4
Rubella	Decrease	−0.5 (−0.8 to −0.1)	< 0.05

A normal (*Z*) distribution was used to assess the significance of the annual percentage change, and the parametric method was used to calculate 95% CIs.

* Results of Cases and APC from pH1N1 were based on the full period from 2009 to 2013.

† Joinpoint program provides significant values as *P* < 0.05.

‡ When incidence data contained zero, we substitute the zero with 1% of the smallest incidence.

For each RID, only seasonal influenza showed a significantly increased trend for the annual mortality rate in these years (*R* = 0.73, *P* = 0.00089, Figure 1B). The other nine RIDs all showed decreased or stable trends, of which avian H5N1 influenza, measles, epidemic cerebrospinal meningitis, mumps, and pH1N1 had significant downward trends (all *P* < 0.05, Figure 1B; Table 3).

Moreover, considering the control measures during the COVID-19 pandemic, including lockdowns, school closures, mask-wearing, and frequent hand washing, might have had an impact on respiratory infectious disease epidemics (26, 27), by comparing the changes in the mortality of each of the 10 RIDs before and after the COVID-19 period (the 5 years before COVID-19 vs. 2020) in China, we found that the overall mortality rate of the 10 RIDs in 2020 decreased by 31.80% (*P* = 0.006). The cerebrospinal meningitis endemic decreased by 75.45% in 2020 compared to the previous 5 years (*P* = 0.045, Table 4). TB decreased by 31.27% in 2020 compared to the previous 5 years (*P* = 0.01, Table 4). More detail is listed in Table 4.

**Table 4:** Changes in number of cases, number of deaths, and mortality for 10 respiratory infectious diseases in 2020 compared to the 5 years before the COVID-19 period in China.

**Diseases**	**Pre-COVID-19 period**		**2020**		**2020 vs. pre-COVID-19 period**	
	**Average yearly no. of cases (average no. of deaths)**	**Average yearly mortality (per 100,000)**	**Average no. of cases (average no. of deaths)**	**Mortality (per 100,000)**	**Changes (%)**	***P*** **-value**
All 10 RIDs	2,227,044 (2,873)	0.208468	1,969,082 (1,995)	0.142167	−31.80	0.006^‡^
TB	826,910 (2,742)	0.198962	670,538 (1,919)	0.136751	−31.27	0.01^†^
Epidemic cerebrospinal meningitis	108 (12)	0.000871	50 (3)	0.000214	−75.45	0.045^†^
Seasonal influenza	1,052,505 (106)	0.007691	1,145,278 (70)	0.004988	−35.14	0.491
pH1N1^*^	0 (0)	0.000000	0 (0)	0.000000	(—)	(—)
Measles	16,008 (12)	0.000871	856 (0)	0.000000	−100.00	0.655
Avian influenza H5N1	2 (1)	0.000073	0 (0)	0.000000	−100.00	0.367
Pertussis	14,944 (2)	0.000145	4,475 (1)	0.000071	−50.90	0.751
Mumps	233,922 (1)	0.000073	129,120 (1)	0.000071	−1.79	0.208
Scarlet fever	72,501 (1)	0.000073	16,564 (1)	0.000071	−1.79	0.208
Rubella	10,149 (1)	0.000073	2,201 (0)	0.000000	−100.00	0.655

Changes = (*x*_1_ – *x*_2_)/*x*_2_ × 100%, *x*_1_: mortality in 2020; *x*_2_: average yearly mortality pre-COVID-19 period (between 2015 and 2019).

“–” means “decreased”; (—), no cases or numbers reported in the system for each infectious disease.

* pH1N1, the full period from 2009 to 2013. The *P*-value was computed through Mann-Whitney *U*-test.

† *P* < 0.05 and^‡^*P* < 0.01.

### Mortality rate ranks of 10 RIDs in China from 2004 to 2020

**Figure 2:**
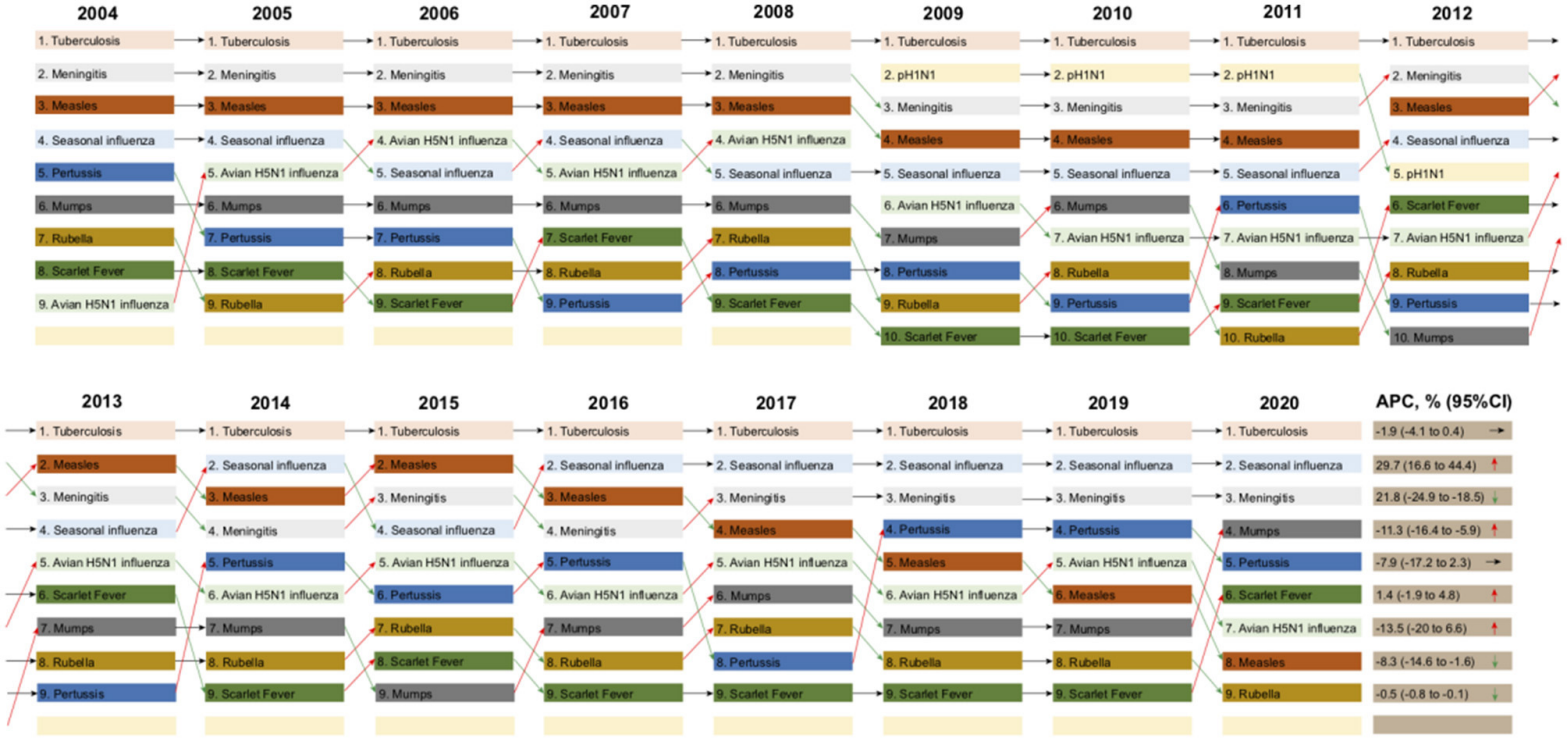
Mortality ranking of each of the 10 respiratory infectious diseases (RIDs) by year, from 2004 to 2020. The annual percentage change (APC) in the mortality of each RID is listed with 95% confidence intervals (CI). Arrows pointing upwards (red) represent overall increased trends in ranking, arrows pointing downwards (green) represent decreased trends in ranking, and arrows pointing to the right (purple) represent stable trends in the mortality rankings of each infectious disease from 2004 to 2020.

As for the relative ranking of these RIDs from 2004 to 2020 (except pH1N1 for the period of 2009–2013), TB was the leading RID in these 17 years, 22.2% (2/9, TB and Pertussis) of the diseases had a stable rank, 44.4% (4/9, Seasonal influenza, Mumps, Scarlet Fever, and Avian H5N1 influenza) had an increased rank, and 33.3% (3/9, Epidemic cerebrospinal meningitis, Measles, and Rubella) had a decreased rank (Figure 2). Tuberculosis, seasonal influenza, and epidemic cerebrospinal meningitis were the three RIDs with the highest mortality rates from 2017 to 2020.

### Geographical distribution in the mortality of RIDs in China from 2004 to 2020

**Figure 3:**
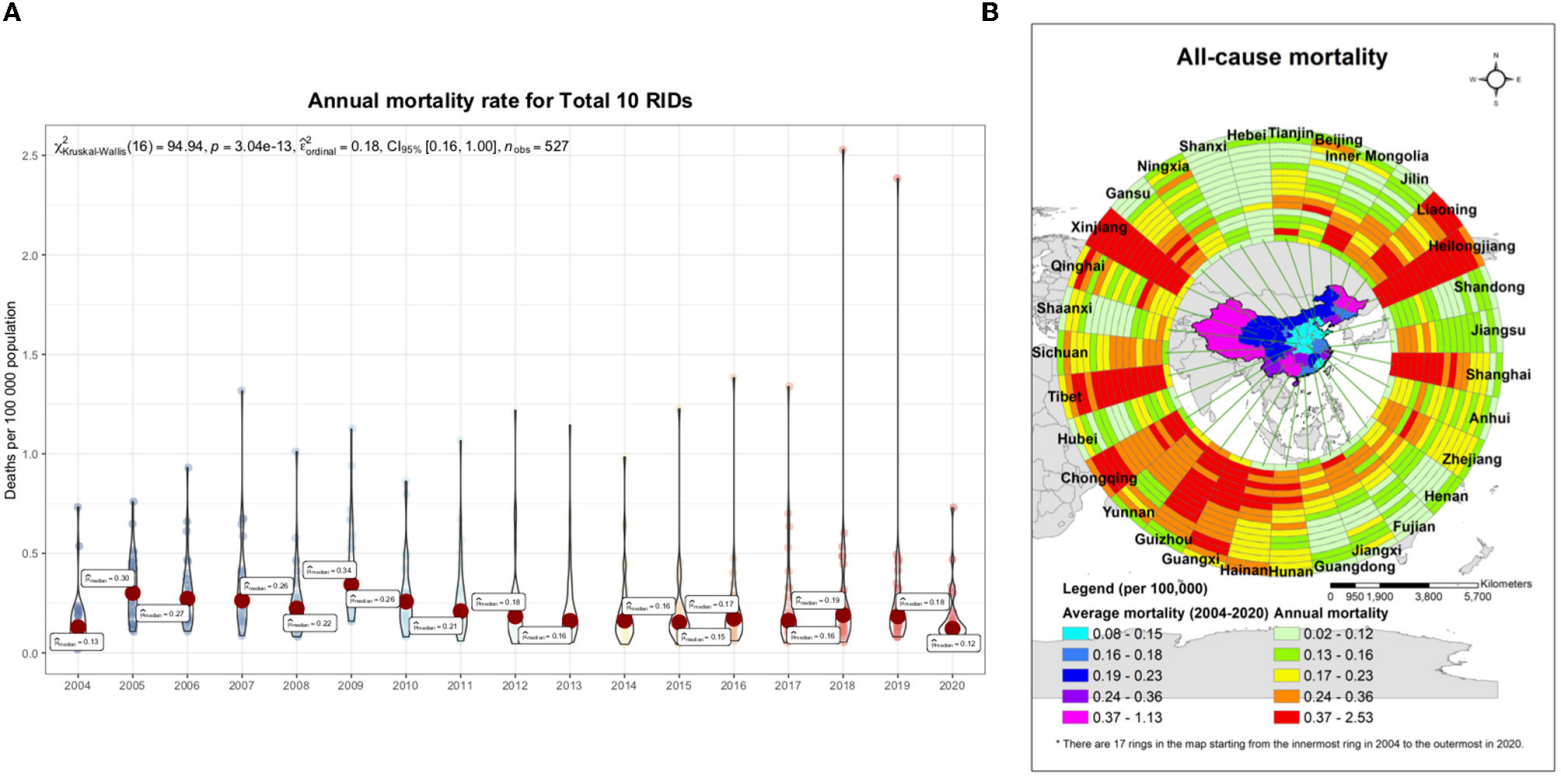
Spatiotemporal distribution of mortality caused by 10 respiratory infectious diseases (RIDs) in 31 province-level units, from 2004 to 2020. **(A)** The epidemic trend for the annual mortality rate from overall respiratory infectious diseases in 31 province-level units, 2004–2020. The small, colored dots indicate the full range across all 31 provinces; and the big red dots indicate the average level. **(B)** The spatiotemporal clusters of overall respiratory infectious diseases. The mortality rate data for all 17 years and 31 provinces were used, with a maximum cluster population size of 10% to minimize false clusters and a maximum temporal window of 3 years to examine the clusters. The local risk ring maps were also done using ArcGIS software. The 14 rings contain data for each year studied, with the innermost ring bearing data for 2004, and moving outwards through the years to the outermost ring, bearing data for 2020.

All provinces reported fatal respiratory cases. However, the average yearly mortality of the 10 diseases was heterogeneously distributed across 31 provinces in mainland China, the northwestern, southwestern, and northeast regions were the highest. The average yearly mortality of all 10 RIDs was highest in Xinjiang, at 1.1309 per 100,000 people, the Tibet Autonomous Region (Hereafter, Tibet) at 0.5896 per 100,000 people, and the Heilongjiang Province, at 0.5109 per 100,000 people (Figures 3A, B).

### Age distribution of deaths, mortality, and percentage of RIDs in China from 2004 to 2020

**Figure 4:**
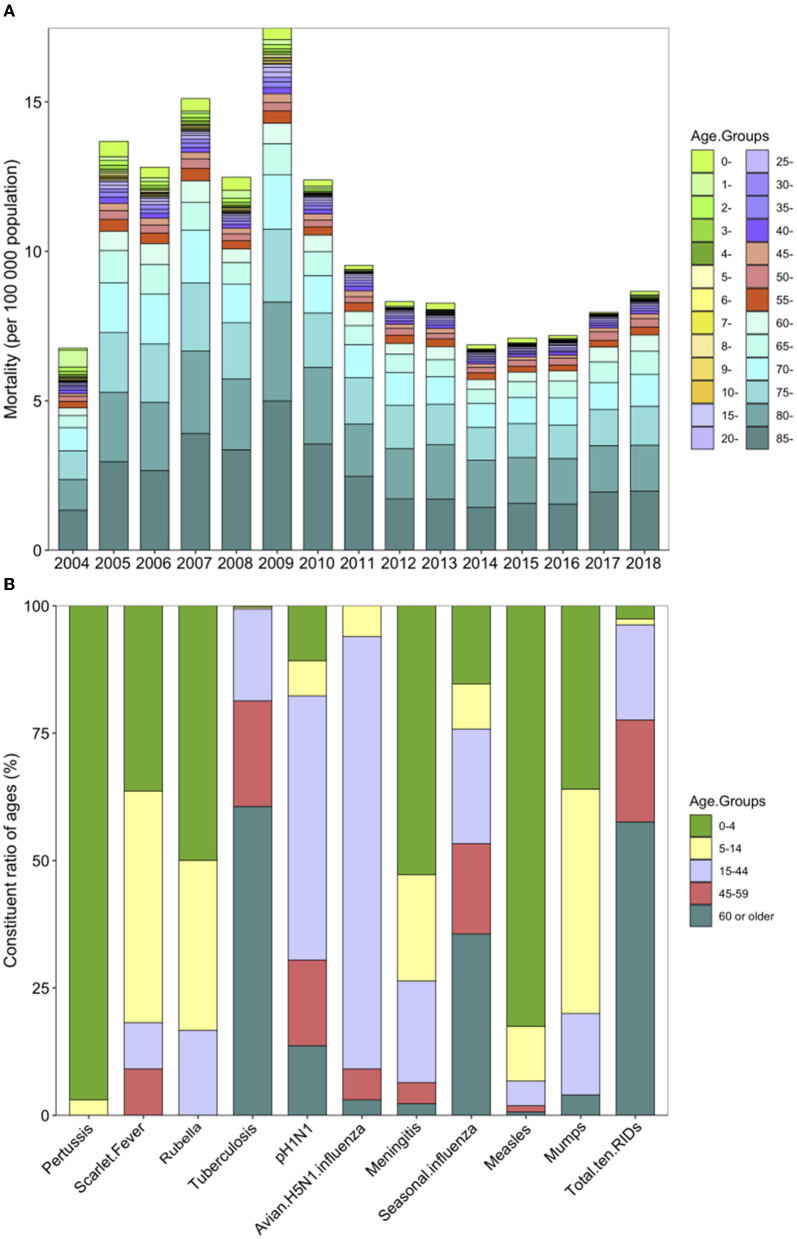
Mortality and constituent ratio of deaths in different ages caused by 10 respiratory infectious diseases (RIDs) in China. **(A)** Mortality (per 100,000 people) due to all 10 RIDs by age group, from 2004 to 2018. **(B)** The constituent ratio of ages in each RID from 2004 to 2020. The colors indicate different age groups; 0–4 years are in green, 5–14 years are in yellow, 15–44 years are in purple, 45–59 years are in red, and 60 years and older are in blue.

Regarding the death incidence rate, the age-specific mortality of the 10 RIDs was highest in people aged 60 or older among all ages from 2004 to 2018, and there has been a decreasing trend in mortality among people aged over 60 since 2010 (Figure 4A). The age group with the highest death constituent ratio was children younger than 5 years old for pertussis, rubella, epidemic cerebrospinal meningitis, and measles from 2004 to 2020 (Figure 4B).

As for the percentage of RIDs, the highest was seen in those aged 70 years or older, accounting for 38.12%, which is 10.16 times those aged 10 years or younger (3.75%; *P* < 0.001) and 2.77 times (38.12 vs. 13.75%; *P* < 0.001) those aged 30 years and above (Supplementary Table 12).

### Case fatality rate of 10 RIDs from 2004 to 2020

**Figure 5:**
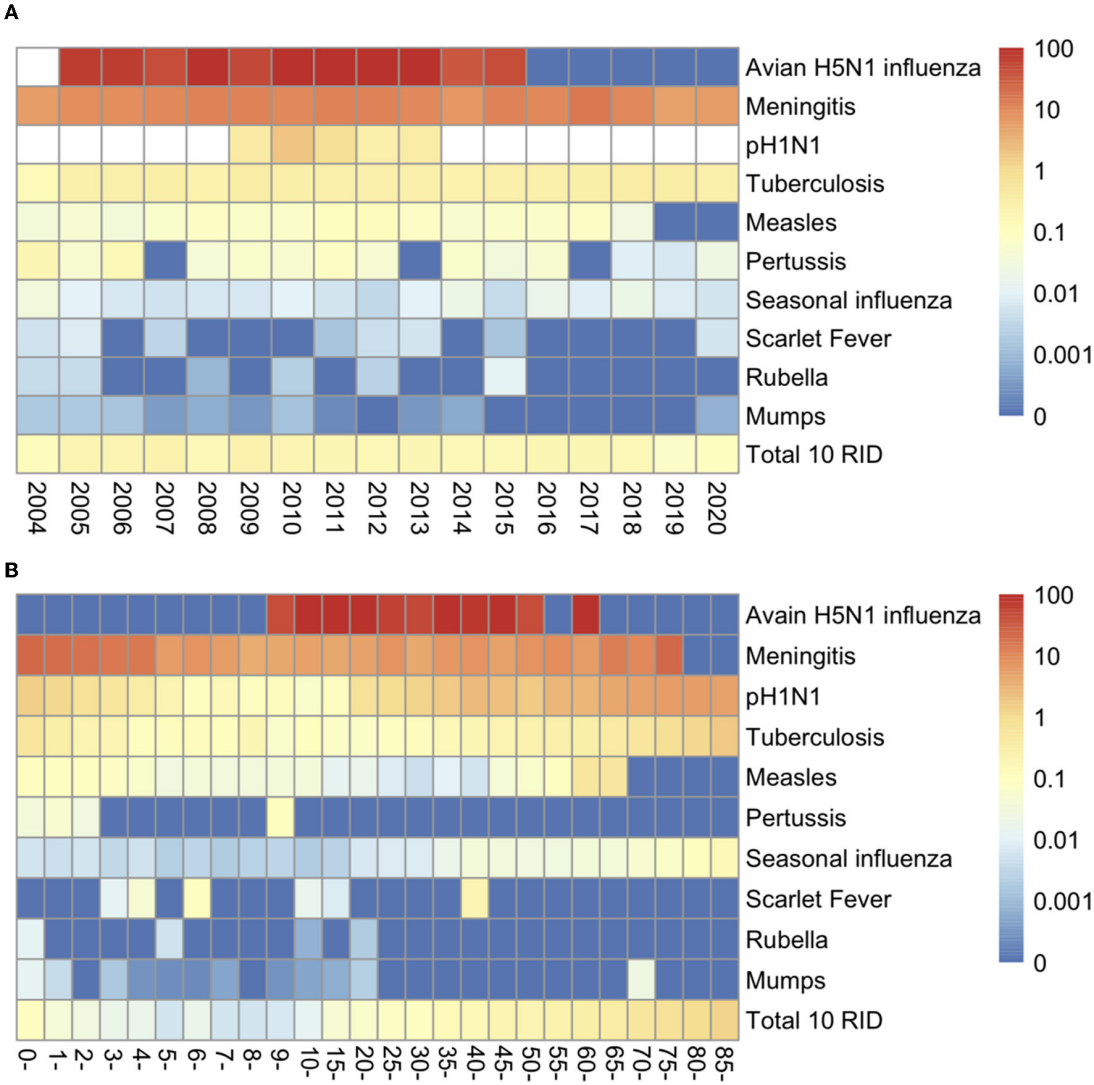
Case Fatality Rates (CFR) for 10 respiratory infectious diseases (RIDs). **(A)** Yearly CFR by year for 2004–2020 in China. **(B)** Age-specific CFR by age groups. Blanks indicate that no reporting data were available.

We analyzed the yearly CFR for the 10 most common causes of respiratory diseases during 2004–2020. The overall CFR was 1.6411 per 1,000 (50,595/30,830,492). The diseases with the highest yearly case fatality ratios were avian influenza A H5N1 [687.5 per 1,000 (33/48)] and epidemic cerebrospinal meningitis [90.5748 per 1,000 (1,010/11,151)] (Table 1; Figure 5A).

The age-specific CFR of the 10 infectious diseases was highest among people older than 85 years and was lowest among children younger than 10 years, particularly in 5-year-olds, with a rate of 13.6551 per 1,000 (2,353/172,316) and 0.0552 per 1,000 (58/1,051,178), respectively (Figure 5B; Supplementary Table 13).

### Tuberculosis

Nationally, the cumulative number of deaths caused by tuberculosis was 47,374 from 2004 to 2020, with 2,787 (95% CI: 2,472–3,101) annual deaths (Table 1). Tuberculosis was the leading cause of respiratory infectious disease mortality from 2004 to 2020 (Figures 1, 2), accounting for 17.22% (47,374/275,071) and 93.63% (47,374/50,595) mortality of all 39 infectious diseases and the 10 respiratory diseases, respectively.

Although TB mortality increased by 33.73% (from 0.1104 in 2004 to 0.1368 in 2020), in 54.84% (17/31) provinces of China, the mortality rate of TB showed a stable trend in the 17 years from 2004 to 2020 (*R* = −0.36, *P* = 0.16, Figure 1). Meanwhile, the Joinpoint regression indicates that the APC of age-adjusted mortality was −1.9% (95% CI −4.1 to 0.4%, *P* = 0.1000) across the whole of China from 2004 to 2020 (Figure 2; Table 3).

TB had the highest absolute inequality among 31 provinces. Developing provinces and areas registered the highest TB mortality rates, namely 1.0751/100,000 in Xinjiang, 0.5488/100,000 in Tibet, and 0.4966/100,000 in the Heilongjiang Province (Supplementary Figure 1).

### Epidemic cerebrospinal meningitis

In mainland China, the cumulative number of deaths caused by epidemic cerebrospinal meningitis was 1,010 from 2004 to 2020, the number of yearly deaths being 59 (95% CI: 25–94) (Table 1). Epidemic cerebrospinal meningitis was the second leading cause of respiratory infectious disease mortality from 2004 to 2020. Epidemic cerebrospinal meningitis only caused 2% (1,010/50,595) of all respiratory disease mortality in China.

The mortality of epidemic cerebrospinal meningitis decreased by 98.18% (from 0.0127 in 2004 to 0.0002 in 2020) in 87.10% (27/31) provinces of China. The mortality rate of epidemic cerebrospinal meningitis showed a significantly decreased trend in the 17 years from 2004 to 2020 (*R* = −0.98, *P* = 1.1e-11, Figure 1). Meanwhile, the Joinpoint regression indicates that the APC of age-adjusted mortality was −21.80% (95% CI −24.9 to −18.5%, *P* < 0.0001) across the whole of China from 2004 to 2020 (Figure 2; Table 3).

During 2004–2020, mortality from epidemic cerebrospinal meningitis was highest in the northwestern and western parts of China, namely in Xinjiang (0.0245/100,000), Tibet (0.0180/100,000), and Beijing (0.0149/100,000) (Supplementary Figure 2).

### Seasonal influenza

Seasonal influenza deaths increased between 2004 and 2020. A total of 711 deaths were caused by seasonal influenza, accounting for 1.41% (711/50,595) of all respiratory infectious deaths; see Table 1. The number of yearly deaths was 42 (95% CI: 6–78) in China, nationwide (Table 1).

At the national level, mortality rates increased by 366.67% between 2004 (0.0012/100,000) and 2020 (0.0050/100,000). 90.32% (28/31) of areas in mainland China reported increased mortality. The mortality rate showed a significantly increased trend in the 17 years from 2004 to 2020 (*R* = 0.73, *P* = 0.00089, Figure 1). Meanwhile, the Joinpoint regression indicates that the APC of age-adjusted mortality was 29.7% (95% CI 16.6–44.4%, *P* < 0.0001) across mainland China from 2004 to 2020 (Figure 2; Table 3).

Across China, influenza mortality was predominantly concentrated in northern and southwestern China, with a mortality rate of 0.0361/100,000 in Beijing, 0.0088/100,000 in Tianjin, and 0.0077/100,000 in Chongqing (Supplementary Figure 3).

### pH1N1

The full period of pH1N1 was from 2009 to 2013; the total deaths caused by pH1N1 accounted for 1.18% (916/50,595) of all respiratory infectious disease deaths during our 17-year study period, while the number of yearly deaths was 183 (95% CI: −149 to 515) in the 5-year period (Table 1).

Overall, the mortality rates decreased by 94.09%, from 0.0491 in 2009 to 0.0029 in 2013 (Table 2; Supplementary Figure 4A). The mortality rate showed a decreased trend in these 5 years, with an APC of −59.4% (95% CI −78.1 to −24.8%, *P* < 0.0001) in China from 2009 to 2013 (Figure 2; Table 3).

From 2009 to 2013, provinces in northwestern, northern, and northeastern China, namely Beijing (0.1087/100,000), Ningxia (0.0325/100,000), and Tianjin (0.0310/100,000) were the areas most affected by pH1N1 (Supplementary Figure 4).

### Avian H5N1 influenza

Avian H5N1 influenza was a zoonotic disease, first identified in China in 2005. The overall deaths caused by H5N1 accounted for 0.07% (33/50,595) of all respiratory infectious disease mortality during our study period (Table 1). The number of annual deaths was 2.1 (95% CI: 0.9–3.3) at the national level.

Overall, the mortality rate showed a significantly decreased trend from 2005 to 2016 (*R* = −0.89, *P* = 4.2e-06, Figure 1). Meanwhile, the Joinpoint regression suggests an APC of −13.5% (95% CI −20 to 6.6%, *P* < 0.0001) in China during this period (Figure 2; Table 3).

The mortality rate of H5N1 varied across 31 provinces, of which, Guizhou Province (0.0007/100,000) and Xinjiang (0.0006/100,000) were the areas most affected by Avian H5N1 influenza (Supplementary Figure 5).

### Measles, mumps, and rubella

Measles caused 0.94% (476/50,595) of all respiratory infectious disease deaths in 2004–2020 (Table 1). Nationally, the measles mortality rate decreased by 100% (from 0.002 in 2004 to 0 deaths per 100,000 individuals in 2020). The mortality rate showed a decreased trend in the 16 years from 2004 to 2020 (*R* = −0.55, *P* = 0.022, Figure 1). Meanwhile, the Joinpoint regression suggests an APC of −8.3% (95% CI −14.6 to 1.6%, *P* < 0.0001), see Table 3. The measles mortality rate was highest in the western part of China, additional agglomeration areas with mortality including Tibet (0.0217 per 100,000 individuals), Xinjiang (0.0173 per 100,000 individuals), and the Qinghai Province (0.0113 per 100,000 individuals); see Supplementary Figure 6.

Mumps caused 0.05% (25/50,595) of all respiratory infectious disease mortality during this period; see Table 1. The mortality rates decreased by 66.67% between 2004 and 2020 (0.0003 vs. 0.0001 deaths per 100,000 individuals) in the Shannxi and Guangxi provinces, see Table 2 and Supplementary Table 14. The mortality rate showed a significantly decreased trend in these 16 years from 2004 to 2020 (*R* = −0.87, *P* = 4.7e-06, Figure 1). Meanwhile, the Joinpoint regression suggests an APC of −11.3% (95% CI −16.4 to −5.9%, *P* < 0.0001) see Table 3.

Rubella caused 0.01% (6/50,595) of all respiratory infectious disease mortality during 2004–2020 (Table 1). The mortality rate showed a significantly decreased trend in the 17 years from 2004 to 2020 (*R* = −0.55, *P* = 0.022, Figure 1) and mortality decreased by −100% between 2004 and 2020 (0.0001 vs. 0 deaths per 100,000 individuals) in the Henan province, see Table 2 and Supplementary Table 15. Meanwhile, the Joinpoint regression suggests an APC of −0.5% (95% CI −0.8 to −0.1%, *P* < 0.0001), see Table 3. Only 5 of 31 provinces recorded deaths caused by rubella (Supplementary Table 15).

### Pertussis

Pertussis accounted for 0.07% (33/50,595) of respiratory infectious disease mortality (Table 1). Although mortality decreased by −85.71% between 2004 and 2020 (0.0007 vs. 0.0001 deaths per 100,000 individuals) in Qinghai, Xinjiang, Chongqing, and the Sichuan province (Table 2), the mortality rate showed a relatively stable trend in the 17 years from 2004 to 2020 (*R* = −0.33, *P* = 0.2, Figure 1). Meanwhile, the Joinpoint regression suggests an APC of −7.9% (95% CI −17.2 to 2.3%, *P* = 0.1) in China from 2004 to 2020 (Table 3). From 2004 to 2008, mortality rates decreased significantly, with an APC of −37.3% (95% CI −56.9 to 8.6%, *P* < 0.0001). Only 14 of 31 provinces reported pertussis deaths in the study period (Supplementary Table 16).

### Scarlet fever

Scarlet fever accounted for 0.02% (11/50,595) of respiratory infectious disease mortality (Table 1). The mortality rate showed a stable trend in the 17 years from 2004 to 2020 (*R* = −0.28, *P* = 0.28, Figure 1). Meanwhile, the Joinpoint regression suggests an APC of 1.4% (95% CI −1.9 to 4.8%, *P* = 0.4) in China from 2004 to 2020 (Table 3). Only eight of 31 provinces reported scarlet fever deaths (Supplementary Table 17).

## Discussion

Based on 17 national mortality statistics, this observational study analyzes the changing patterns and trends in the mortality rates of 10 RIDs in China (28–30). We found a relatively stable trend in the mortality rate caused by all 10 RIDs from 2004 to 2020. Moreover, with the exception of an increase in mortality for seasonal influenza, the other RIDs showed a decreased or stable trend. With the continuous containment and mitigation strategies for COVID-19 in 2020, China has significantly reduced the overall mortality for the 10 RIDs compared to the previous 5 years. Both levels of mortality and rates of change varied by region, age group, and subcategories of respiratory causes in the past 17 years. To our knowledge, this is the first study to report the trends and yearly mortality rates of 10 RIDs from 2004 to 2020 in China.

Similar to the USA and UK reports, this study indicates that the overall mortality rates of the 10 infectious diseases have shown a striking decline in China since 2009 (2, 4). The sharp reduction is not well-understood, but it could be attributed to a multiplicity of factors. First, comprehensive prevention and control measures for infectious diseases in terms of resources, systems, and laws since the SARS endemic in 2003 in China have contributed to this decreasing trend, such as the 2003 Emergency Regulations for Public Health Emergencies, Law of the People's Republic of China on prevention and control of infectious diseases in 2004, 2013, and 2020 (31). Importantly, NPIs for the prevention of COVID-19 have contributed to the reduction in RID mortality in China. Second, large-scale vaccination (22 National Immunization Program vaccines increased from 78% in 2005–2007 to ≥98% in 2015) and the development of effective treatments may have also contributed to this decline. Third, the Chinese government increased investments to US$2.25 billion to control infectious diseases starting in 2008 and started a national science project to end tuberculosis and measles in recent years (9, 32). Fourth, the diagnosis of infectious diseases has improved gradually in recent decades, in particular, PCR (polymerase chain reaction) rapid diagnosis has been widely adopted at all levels of hospitals (9, 33). Early diagnosis and proper case management of these cases can increase the survival rate. Fifth, China experienced improved air quality during this period, where the pollution level and the number of extremely polluted days have decreased substantially (34). Lastly, improved living standards, particularly, improved hygiene and personal nutrition, may have also contributed to this decreasing trend (9).

Our study indicates that the tuberculosis mortality rate declined significantly from 2004 to 2020, which means China will be much closer to the SDG TB targets by 2030 (35). This is in line with previous studies. There are several factors that explain these changes. On the one hand, in 2005, China rebuilt public health service facilities and formulated a new 5-year plan for tuberculosis prevention and treatment (36). On the other hand, living standards, health services, medical technology, and the monitoring of infectious diseases have improved in the past 10 years (37).

The influenza-associated respiratory mortality rate estimates from 1999 to 2015 are higher than previously reported globally (38, 39). In China, the seasonal influenza mortality rate had a relatively high increasing trend from 2004 to 2020, in particular, a sudden rise from 2017 to 2019. The reason for this increasing trend was not clear but could be attributed to pathogen shift and low vaccination coverage (21, 40, 41). As to the former, seasonal influenza saw a large peak in these 2 years because B/Yamagata was the main epidemic strain, but this type had not emerged for the previous 2 years, so the population had no antibodies (21); secondly, the seasonal influenza vaccine is not included in the National Immunization Program, so the average national vaccination rate was just 1.5–2.2% between 2004 and 2014 (42). Noticeably, the diagnosis standard of confirmed influenza cases was changed in 2019, and the influenza antigen rapid test is now included in the diagnosis guidelines for influenza-confirmed cases according to the national diagnostic and treatment protocol for seasonal influenza (2019 edition). This is a potential contributing factor in the increased mortality of seasonal influenza.

We have observed notable geographic patterns in the mortality rates for respiratory infectious diseases, with substantial increases in some of the undeveloped regions of western, northwestern, and northeastern China, namely, Xinjiang, the Tibet Autonomous Region, and the Heilongjiang Province. These differences were likely driven by the climate, demographic characteristics, pathogen subtypes, vaccination, public health emergencies, personal protection and vaccination, and environmental sanitation. Firstly, lower reported coverage rates (<90%) and poor medical availability were found in western areas (9, 40). Other important factors were less efficient public health systems, the multiethnic population, poverty, heavy smoking, and poor sanitation (9, 43, 44). Finally, the respiratory disease mortality rate in northern China is positively associated with air pollution according to published articles (34). The effects of air pollution are greatest in northern cities during cold months, when coal is burned for heating (13, 34).

The age pattern in the fatal cases among the 10 different diseases is similar to those observed in other reports (38, 45). The aging population of China might have contributed to higher mortality rates of primary respiratory diseases, namely TB and influenza. The drift of TB and seasonal influenza to the population over 75 may be triggered by the growing elderly population in China (46). Life expectancy increased from 68.55 years in 1990 to 76.34 years in 2015, and this significant increase in life expectancy adds significantly to the disease burden in China (22). There is a need to develop national strategies, including health care, personal and social care, and economic support to improve the health of the aging population (47). Low non-immunization program vaccination rate, comorbidity of chronic diseases, drug resistance, and untimely treatment may be possible reasons for the high mortality rate of respiratory disease among the elderly (48). Moreover, the overall yearly mortality rate of the other eight respiratory diseases was higher among children under 15 years old, in particular, those below 5 years old. Due to the recent introduction of the third-child policy, we anticipate an increase of 1–2 million more births every year (22). Increased capacity in child health services, the national vaccination program, and health insurance systems are needed to address the needs of the increasing child population (49).

The CFR for the 10 RIDs during the study period was much lower than that reported in the Yang et al. report in 2013 (9). The RIDs with the highest mortality rates in terms of yearly CFR were avian influenza H5N1, epidemic cerebrospinal meningitis, and pH1N1. The severity of influenza and avian influenza might vary depending on the disease subtype. CFR in high-pathogenicity H5N1 (70%) was far higher than in low-pathogenicity avian influenza (H7N9, 40.4%) in China (50). However, the seasonal influenza-associated CFR rate (0.0638 per 1,000) was very low compared with avian influenza epidemics. Generally, the overall CFR for avian influenza and seasonal influenza in China was higher than the global average [53.5% for H5N1 (51) and 0.24% for pH1N1 (52)]. This increased rate may be the result of several factors. On the one hand, diagnosis of the H5N1 infection was delayed because H5N1 was screened by the surveillance results of unexplained pneumonia cases. On the other hand, most H5N1 cases were found in poor and rural areas (51, 53), where early diagnosis is difficult. The CFR of epidemic cerebrospinal meningitis in the study period in China was much lower than that in Nigeria (13.8%) and the sub-Saharan epidemic cerebrospinal meningitis belt (9.3%) (54), but similar to that in Ghana (5.8%) (55). This may be due to increased vaccination based on the Expanded Program on Immunization (EPI) and consequent herd immunity of the child population in China. However, the CFR for epidemic cerebrospinal meningitis in China is still very high, suggesting the need for a more coordinated approach aimed at improving disease notification, early diagnosis, and effective treatment (56).

The mortality rate of these RIDs imposes a significant economic burden. For example, a course of treatment for TB in China costs ~US$149 to 724, representing around 42–119% of the average household income (57). This overall cost is likely to increase in the future due to the aging and growth of the population. It is important to discuss what measures should be taken to further decrease the disease burden that poses a great public health challenge. We would suggest, first of all, that we need to improve the timeliness of diagnosis by developing newer technology in laboratory detection (9). Second, the government needs to improve the availability and quality of healthcare systems, especially in the western and northern regions. Third, it is important to increase immunization coverage in the child and elderly population for vaccine-preventable diseases, in particular, influenza (39, 40, 58). Fourth, developing newer antibiotics and vaccines should also be a priority measure to improve clinical outcomes (4).

This study still has several limitations. First, we cannot analyze the mortality rate stratified by suspected, clinically diagnosed, and confirmed cases. Second, there was no seasonal influenza death surveillance system in China, and specimens for the detection of influenza were not taken in every instance of death in an influenza-like illness case. Seasonal influenza mortality could be underestimated. Third, without data on the population age groups in 2019–2020, we can only analyze the mortality rate from 2004 to 2018. Fourth, the comparability of data in 2020 with data in previous years may have been affected by the COVID-19 pandemic (26, 27, 59). Nevertheless, we performed sensitivity analyses (using data from 2004 to 2019) and the results remained stable. Due to the unavailability of data from 2021 to 2022, the impact of the COVID-19 epidemic on the morbidity and mortality rates of other infectious diseases cannot be assessed. We will continue to carry out in-depth research in the future.

## Conclusions

Our study shows that the yearly mortality rates for all respiratory diseases were perennially stable in China from 2004 to 2020. However, the overall mortality rates of the 10 RIDs during the initial outbreak of COVID-19 were lower than in the previous 5 years. Between 2004 and 2020, there were significant differences in mortality rates and changes in mortality by province, age, and respiratory disease type. The highest burden of annual respiratory deaths was observed in the undeveloped western, northwestern, and northern parts of China. Those aged 60 or above and children aged five and below are at risk of death. TB represents the largest burden among overall respiratory disease deaths in China, and the greatest increasing mortality rate trend was observed in seasonal influenza. These estimates may be helpful to provincial and national policymakers in the improvement of prevention, diagnosis, and treatment strategies for respiratory diseases, especially the expansion of vaccination programs, improved implementation, and increased government financing.

## Data availability statement

ADPKD-affected monkeys' genome sequence reads from this study have been deposited in the Sequence Read Archive (SRA) database maintained by the National Center for Biotechnology Information (NCBI) (Accession Nos. SUB10161089, PRJNA752494, and SRP333006). WGS raw sequence data of wildtype monkeys used for comparative analysis in this article have been deposited in the Genome Sequence Archive (42, 59) in the BIG Data Center (Nucleic Acids Res 2018), Beijing Institute of Genomics (BIG), Chinese Academy of Sciences, under accession number CRA002684, and are publicly accessible at https://bigd.big.ac.cn/gsa.

## Author contributions

ZZ, SL, T-CC, and NZ conceived the study design. NZ collected the data, performed the field investigation, and drafted the manuscript. NZ, SW, and LW developed statistical models and data analysis. YS, YJ, and T-JT collected the data and performed the field investigation. All authors listed contributed to and approved the final results, manuscript drafts, and this publication.

## References

[1] GBD 2017 DALYs and HALE Collaborators. Global, regional, and national disability-adjusted life-years (DALYs) for 359 diseases and injuries and healthy life expectancy (HALE) for 195 countries and territories, 1990-2017: a systematic analysis for the Global Burden of Disease Study 2017. Lancet. (2018) 392:1859–922. 10.1016/S0140-6736(18)32335-3 30415748 PMC6252083

[2] Salciccioli JD Marshall DC Shalhoub J Maruthappu M De Carlo G Chung KF. Respiratory disease mortality in the United Kingdom compared with EU15+ countries in 1985-2015: observational study. BMJ. (2018) 363:k4680. 10.1136/bmj.k4680 30487157 PMC6259045

[3] GBD 2016 Causes of Death Collaborators. Global, regional, and national age-sex specific mortality for 264 causes of death, 1980-2016: a systematic analysis for the Global Burden of Disease Study 2016. Lancet. (2017) 390:1151–210. 10.1016/S0140-6736(17)32152-9 28919116 PMC5605883

[4] El Bcheraoui C Mokdad AH Dwyer-Lindgren L Bertozzi-Villa A Stubbs RW Morozoff C. Trends and patterns of differences in infectious disease mortality among US counties, 1980-2014. JAMA. (2018) 319:1248–60. 10.1001/jama.2018.2089 29584843 PMC5885870

[5] Caini S Spreeuwenberg P Kusznierz GF Rudi JM Owen R Pennington K. Distribution of influenza virus types by age using case-based global surveillance data from twenty-nine countries, 1999–2014. BMC Infect Dis. (2018) 18:1–10. 10.1186/s12879-018-3181-y 29884140 PMC5994061

[6] Zhou Y Chen C Jiang H Pan HQ Zhu LM Lu W. High admission rates and heavy inpatient service costs of urban tuberculosis patients in eastern China. BMC Health Serv Res. (2019) 19:1–12. 10.1186/s12913-019-3892-9 30658635 PMC6339337

[7] Liu Q Smith H Wang Y Tang SL Wang QL Garner P. Tuberculosis patient expenditure on drugs and tests in subsidised, public services in China: a descriptive study. Trop Med Int Health. (2010) 15:26–32. 10.1111/j.1365-3156.2009.02427.x 19917035

[8] Jiang Y Dou X Yan C Wan L Liu H Li M. Epidemiological characteristics and trends of notifiable infectious diseases in China from 1986 to 2016. J Glob Health. (2020) 10:020803. 10.7189/jogh.10.020803 33214900 PMC7649044

[9] Yang S Wu J Ding C Cui Y Zhou Y Li Y. Epidemiological features of and changes in incidence of infectious diseases in China in the first decade after the SARS outbreak. Lancet Infect Dis. (2017) 17:716–25. 10.1016/S1473-3099(17)30227-X 28412150 PMC7164789

[10] Du M Wang R Yuan J Lv X Yan W Liu Q. Trends and disparities in 44 national notifiable infectious diseases in China. An analysis of national surveillance data from 2010 to 2019. J Med Virol. (2022) 95:e28353. 10.1002/jmv.28353 36443103 PMC10107249

[11] Liu Q Qin C Du M Wang Y Yan W Liu M. Incidence and mortality trends of upper respiratory infections in China and other Asian Countries from 1990 to 2019. Viruses. (2022) 14:2550. 10.3390/v14112550 36423159 PMC9697955

[12] Zignol M Hosseini MS Wright A Lambregts-van Weezenbeek C Nunn P Watt CJ. Global incidence of multidrug-resistant tuberculosis. J Infect Dis. (2006) 194:479–85. 10.1086/505877 16845631

[13] Liu YH Chan TC Yap LW Luo YP Xu WJ Qin SW. Resurgence of scarlet fever in China: a 13-year population-based surveillance study. Lancet Infect Dis. (2018) 18:903–12. 10.1016/S1473-3099(18)30231-7 29858148 PMC7185785

[14] Chen Y Zhao Y. Multidrug-resistant tuberculosis in rural China: lack of public awareness, unaffordable costs and poor clinical management. BMJ Case Rep. (2018) 2018:1–4. 10.1136/bcr-2018-225794 30100573 PMC6088315

[15] Zhang Y Li L Dong XC Kong M Gao L Dong XJ. Influenza surveillance and incidence in a rural area in China during the 2009/2010 influenza pandemic. PLoS ONE. (2014) 9:115347. 10.1371/journal.pone.0115347 25542003 PMC4277345

[16] Li Q Zhou L Zhou MH Chen ZP Li FR Wu HY. Epidemiology of human infections with avian influenza A(H7N9) virus in China. New Engl J Med. (2014) 370:520–32. 10.1056/NEJMoa1304617 23614499 PMC6652192

[17] Zhou L Tan Y Kang M Liu FQ Ren RQ Wang YL. Preliminary epidemiology of human infections with highly pathogenic avian influenza A(H7N9) virus, China, 2017. Emerg Infect Dis. (2017) 23:1355–9. 10.3201/eid2308.170640 28580900 PMC5547798

[18] Pan M Gao RB Lv Q Huang SH Zhou ZH Yang L. Human infection with a novel, highly pathogenic avian influenza A (H5N6) virus: virological and clinical findings. J Infection. (2016) 72:52–9. 10.1016/j.jinf.2015.06.009 26143617

[19] Yang ZF Mok CKP Peiris JSM Zhong NS. Human infection with a novel avian influenza A(H5N6) virus. New Engl J Med. (2015) 373:487–9. 10.1056/NEJMc1502983 26222578

[20] Bedford T Riley S Barr IG Broor S Chadha M Cox NJ. Global circulation patterns of seasonal influenza viruses vary with antigenic drift. Nature. (2015) 523:217–U06. 10.1038/nature14460 26053121 PMC4499780

[21] Yang J Lau YC Wu P Feng LZ Wang XL Chen T. Variation in influenza B virus epidemiology by lineage, China. Emerg Infect Dis. (2018) 24:1536–40. 10.3201/eid2408.180063 30015611 PMC6056115

[22] Ouyang Y. China relaxes its one-child policy. Lancet. (2013) 382:e28. 10.1016/S0140-6736(13)62544-1 24298609

[23] Zeng Y Cao Y Qiao X Seyler BC Tang Y. Air pollution reduction in China: recent success but great challenge for the future. Sci Total Environ. (2019) 663:329–37. 10.1016/j.scitotenv.2019.01.262 30711599

[24] Dong Y Wang L Burgner DP Miller JE Song Y Ren X. Infectious diseases in children and adolescents in China: analysis of national surveillance data from 2008 to 2017. BMJ. (2020) 369:m1043. 10.1136/bmj.m1043 32241761 PMC7114954

[25] Kulldorff M Athas WF Feurer EJ Miller BA Key CR. Evaluating cluster alarms: a space-time scan statistic and brain cancer in Los Alamos, New Mexico. Am J Public Health. (1998) 88:1377–80. 10.2105/AJPH.88.9.1377 9736881 PMC1509064

[26] Wang L Wang K Zhong H Zhao N Xu W Yang Y. The effect of coronavirus 2019 disease control measures on the incidence of respiratory infectious disease and air pollutant concentrations in the Yangtze River Delta Region, China. Int J Environ Res Public Health. (2022) 19:1286. 10.3390/ijerph19031286 35162304 PMC8835036

[27] Bai BK Jiang QY Hou J. The COVID-19 epidemic and other notifiable infectious diseases in China. Microbes Infect. (2022) 24:104881. 10.1016/j.micinf.2021.104881 34419605 PMC8375246

[28] Notice to Readers. Final 2015 Reports of Nationally Notifiable Infectious Diseases and Conditions. MMWR Morb Mortal Wkly Rep. (2016) 65:1306–21. 10.15585/mmwr.mm6546a9 27880744

[29] Centers for Disease Control and Prevention. Notice to readers: final 2012 reports of nationally notifiable infectious diseases. MMWR Morb Mortal Wkly Rep. (2013) 62:669–82. Available online at: https://pubmed.ncbi.nlm.nih.gov/24133698/24133698 PMC4604800

[30] Centers for Disease Control and Prevention. Notice to readers: final 2013 reports of nationally notifiable infectious diseases. MMWR Morb Mortal Wkly Rep. (2014) 63:702–15. Available online at: https://www.cdc.gov/mmwr/preview/mmwrhtml/mm6332a6.htm 25272402 PMC4584913

[31] Yang W. Dramatic achievements in infectious disease prevention and treatment in China during the past 70 years. Zhonghua Liuxingbingxue Zazhi. (2019) 40:1493–8. 10.3760/cma.j.issn.0254-6450.2019.12.001 32062906

[32] Floyd K Glaziou P Zumla A Raviglione M. The global tuberculosis epidemic and progress in care, prevention, and research: an overview in year 3 of the End TB era. Lancet Respir Med. (2018) 6:299–314. 10.1016/S2213-2600(18)30057-2 29595511

[33] Zhang H Wang L Lai S Li Z Sun Q Zhang P. Surveillance and early warning systems of infectious disease in China: from 2012 to 2014. Int J Health Plann Manage. (2017) 32:329–38. 10.1002/hpm.2434 28632912

[34] Zhou M He G Liu Y Yin P Li Y Kan H. The associations between ambient air pollution and adult respiratory mortality in 32 major Chinese cities, 2006-2010. Environ Res. (2015) 137:278–86. 10.1016/j.envres.2014.12.016 25601729

[35] Chin DP. The COVID-19 pandemic and elimination of tuberculosis in China. China CDC Wkly. (2021) 3:260–4. 10.46234/ccdcw2021.069 34594862 PMC8392955

[36] Wang L Liu J Chin DP. Progress in tuberculosis control and the evolving public-health system in China. Lancet. (2007) 369:691–6. 10.1016/S0140-6736(07)60316-X 17321314 PMC7134616

[37] Cui Y Shen H Wang F Wen H Zeng Z Wang Y. A long-term trend study of tuberculosis incidence in China, India and United States 1992-2017: a joinpoint and age-period-cohort analysis. Int J Environ Res Public Health. (2020) 17:1–19. 10.3390/ijerph17093334 32403353 PMC7246898

[38] Iuliano AD Roguski KM Chang HH Muscatello DJ Palekar R Tempia S. Estimates of global seasonal influenza-associated respiratory mortality: a modelling study. Lancet. (2018) 391:1285–300. 10.1016/S0140-6736(17)33293-2 29248255 PMC5935243

[39] Sullivan SG Cowling BJ. Reconciling estimates of the global influenza burden. Lancet Respir Med. (2019) 7:8–9. 10.1016/S2213-2600(18)30511-3 30553846

[40] Xu L Qin Y Yang J Han W Lei Y Feng H. Coverage and factors associated with influenza vaccination among kindergarten children 2-7 years old in a low-income city of north-western China (2014-2016). PLoS ONE. (2017) 12:e0181539. 10.1371/journal.pone.0181539 28749980 PMC5531459

[41] Ye C Zhu W Yu J Li Z Hu W Hao L. Low coverage rate and awareness of influenza vaccine among older people in <city>Shanghai</city>, China: a cross-sectional study. Hum Vaccin Immunother. (2018) 14:2715–21. 10.1080/21645515.2018.1491246 29995561 PMC6314411

[42] Yang J Atkins KE Feng L Pang M Zheng Y Liu X. Seasonal influenza vaccination in China: landscape of diverse regional reimbursement policy, and budget impact analysis. Vaccine. (2016) 34:5724–35. 10.1016/j.vaccine.2016.10.013 27745951

[43] Wang L Wang Y Jin S Wu Z Chin DP Koplan JP. Emergence and control of infectious diseases in China. Lancet. (2008) 372:1598–605. 10.1016/S0140-6736(08)61365-3 18930534 PMC7138027

[44] Wu J Yang Y. Inequality trends in the demographic and geographic distribution of health care professionals in China: data from 2002 to 2016. Int J Health Plann Manag. (2019) 34:e487–508. 10.1002/hpm.2664 30238482

[45] Gao L Lu W Bai L Wang X Xu J Catanzaro A. Latent tuberculosis infection in rural China: baseline results of a population-based, multicentre, prospective cohort study. Lancet Infect Dis. (2015) 15:310–9. 10.1016/S1473-3099(14)71085-0 25681063

[46] Zhu S Xia L Yu S Chen S Zhang J. The burden and challenges of tuberculosis in China: findings from the global burden of disease study 2015. Sci Rep. (2017) 7:14601. 10.1038/s41598-017-15024-1 29097809 PMC5668247

[47] Li X Lu J Hu S Cheng KK De Maeseneer J Meng Q. The primary health-care system in China. Lancet. (2017) 390:2584–94. 10.1016/S0140-6736(17)33109-4 29231837

[48] Cheng Y Li J Peng Z Zhang M Qin Y Yang X. Analysis on prevention and control of some infectious diseases in the elderly aged 60 years and above in China and countermeasure recommendation. Zhonghua liu xing bing xue za zhi. (2021) 42:28–32. 10.3760/cma.j.cn112338-20200812-01063 33503695

[49] Editorial. The two-child policy in China: what to expect? Lancet. (2013) 382:1758. 10.1016/S0140-6736(13)62534-9 24290582

[50] Wu ZQ Zhang Y Zhao N Yu Z Pan H Chan TC. Comparative epidemiology of human fatal infections with novel, high (H5N6 and H5N1) and low (H7N9 and H9N2) pathogenicity avian influenza A viruses. Int J Env Res Pub He. (2017) 14:1–20. 10.3390/ijerph14030263 28273867 PMC5369099

[51] Lai SJ Qin Y Cowling BJ Ren X Wardrop NA Gilbert M. Global epidemiology of avian influenza A H5N1 virus infection in humans, 1997-2015: a systematic review of individual case data. Lancet Infect Dis. (2016) 16:E108–18. 10.1016/S1473-3099(16)00153-5 27211899 PMC4933299

[52] Doshi SS Stauffer KE Fiebelkorn AP Lafond KE Davidson HA Apostolou A. The burden and severity of illness due to 2009 pandemic influenza A (H1N1) in a large us city during the late summer and early fall of 2009. Am J Epidemiol. (2012) 176:519–26. 10.1093/aje/kws137 22952308

[53] Cowling BJ Jin LM Lau EHY Liao QH Wu P Jiang H. Comparative epidemiology of human infections with avian influenza A H7N9 and H5N1 viruses in China: a population-based study of laboratory-confirmed cases. Lancet. (2013) 382:129–37. 10.1016/S0140-6736(13)61171-X 23803488 PMC3777567

[54] Borrow R Alarcon P Carlos J Caugant DA Christensen H Debbag R. The Global Meningococcal Initiative: global epidemiology, the impact of vaccines on meningococcal disease and the importance of herd protection. Expert Rev Vaccines. (2017) 16:313–28. 10.1080/14760584.2017.1258308 27820969

[55] Kwarteng A Amuasi J Annan A Ahuno S Opare D Nagel M. Current meningitis outbreak in Ghana: historical perspectives and the importance of diagnostics. Acta Trop. (2017) 169:51–6. 10.1016/j.actatropica.2017.01.014 28122199

[56] Li JH Shao ZJ Liu G Bai XL Borrow R Chen M. Meningococcal disease and control in China: findings and updates from the global meningococcal initiative (GMI). J Infection. (2018) 76:429–37. 10.1016/j.jinf.2018.01.007 29406154

[57] Long Q Smith H Zhang TH Tang SL Garner P. Patient medical costs for tuberculosis treatment and impact on adherence in China: a systematic review. BMC Public Health. (2011) 11:1–9. 10.1186/1471-2458-11-393 21615930 PMC3125370

[58] Wang Q Yue N Zheng MY Wang DL Duan CX Yu XG. Influenza vaccination coverage of population and the factors influencing influenza vaccination in mainland China: a meta-analysis. Vaccine. (2018) 36:7262–9. 10.1016/j.vaccine.2018.10.045 30340886

[59] Wang L Guo X Zhao N Ouyang Y Du B Xu W. Effects of the enhanced public health intervention during the COVID-19 epidemic on respiratory and gastrointestinal infectious diseases in China. J Med Virol. (2022) 94:2201–11. 10.1002/jmv.27619 35067944 PMC9015532

