# Supplementary material for: Epidemiological features and trends in the mortality rates of 10 notifiable respiratory infectious diseases in China from 2004 to 2020: Based on national surveillance
Source: Front Public Health. 2023 Feb 17;11:1102747. doi: 10.3389/fpubh.2023.1102747 (PMC9982089; doi:10.3389/fpubh.2023.1102747)
Supplement: Supplementary file 1 [file Data_Sheet_1.PDF]

Supplementary Files

Joinpoint regression

Joinpoint regression analysis fits a series of joined linear models of the natural logarithm of annual incidence using calendar year as an independent variable (Kim HJ, Fay MP, Feuer EJ, Midthune DN. Permutation tests for joinpoint regression with applications to cancer rates. *Stat Med* 2000; **19:** 335–51), which is often useful to describe changes in trend data.

The joinpoint regression model for the observations ($x1$, y1),..., (xn , yn),where $x1$ ≤ ... ≤ xn without loss of generality, may be written as

$$+ + E[y \mid x] = \beta 0 + \beta 1x + \delta 1(x - \tau 1) + ... + \delta k\ (x - \tau k\ )$$

where y is the outcome of interest, x is the calendar year, the τk 's are the unknown joinpoints and $\alpha + = \alpha$ for$\alpha > 0$ and 0 otherwise.

We allowed a maximum of 5 joinpoints for estimation as suggested by the program developers and used Bayesian information criterion (BIC) to select the best-fitted model. Once the unknown joinpoints τk was determined, we estimate the annual percent of changes (APCs) of each period segment18; the APC is calculated as APCi = [(Exp(βi) - 1)] × 100, where βi represents the slope of the period segment19. We also estimated the average annual percent change (AAPC) assuming there is only one segment for the full range of our study periods. Joinpoint regression analysis was conducted with joinpoint Regression Program version 4.1.1 (Statistical Research and Applications Branch, National Cancer Institute).[1]

Reference:

1. Liu Y, Chan TC, Yap LW, Luo Y, Xu W, Qin S, et al. **Resurgence of scarlet fever in China: a 13-year population-based surveillance study**. *Lancet Infect Dis* 2018; 18(8):903-912.

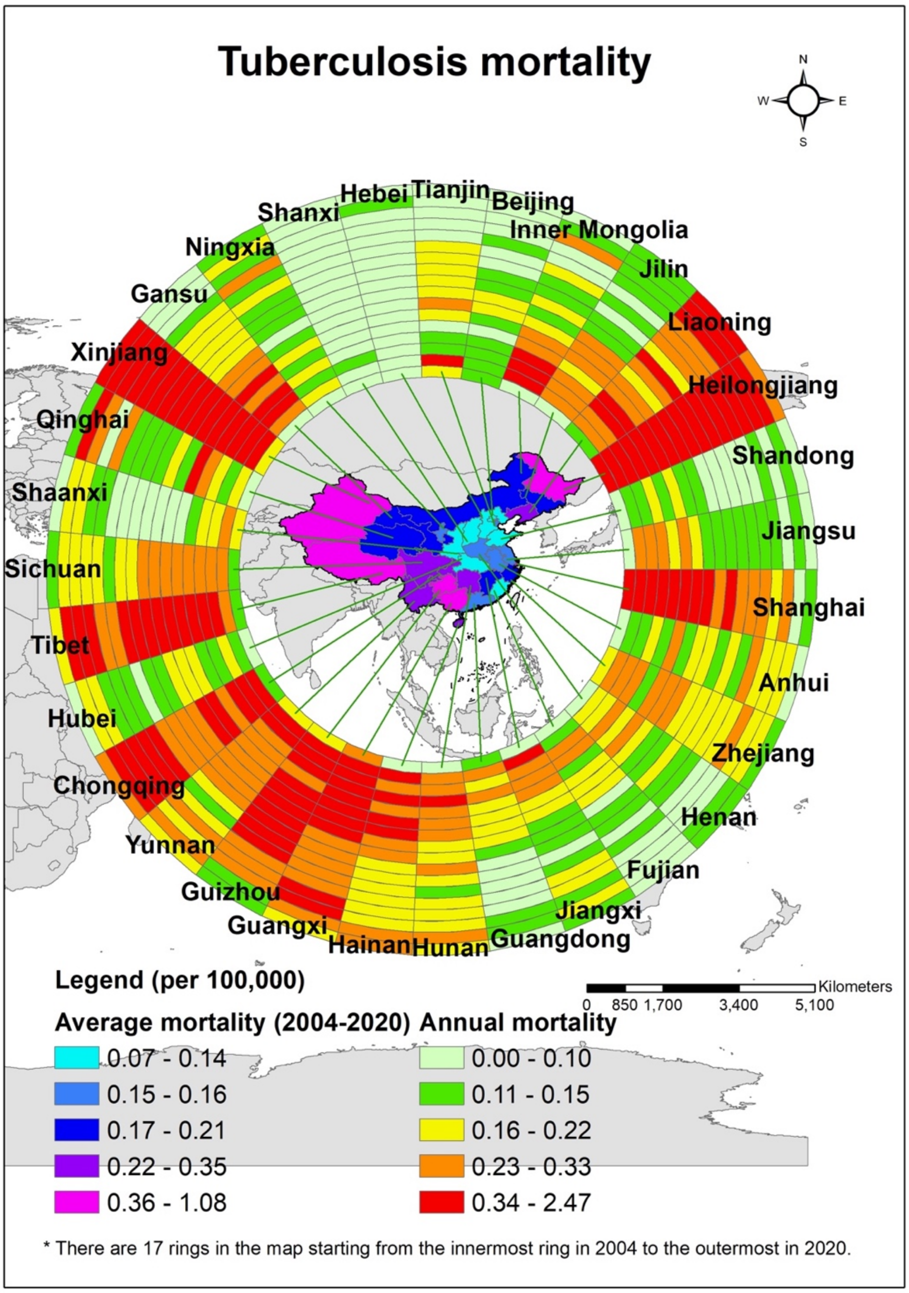

Supplementary Figure 1. Spatiotemporal distribution of the mortality due to Tuberculosis (TB) in 31 province-level units, from 2004 to 2020.

The spatiotemporal clusters of TB. All the 17 years' mortality data for 31 provinces were used, with a maximum cluster population size of 10% to minimize false clusters, and a maximum temporal window of three years to examine the clusters. The local risk ring maps were also done by the ArcGIS software. The 14 rings contain data for each year studied, with the innermost ring bearing data for 2004, and moving outwards through the years to the outermost ring bearing data for 2020.

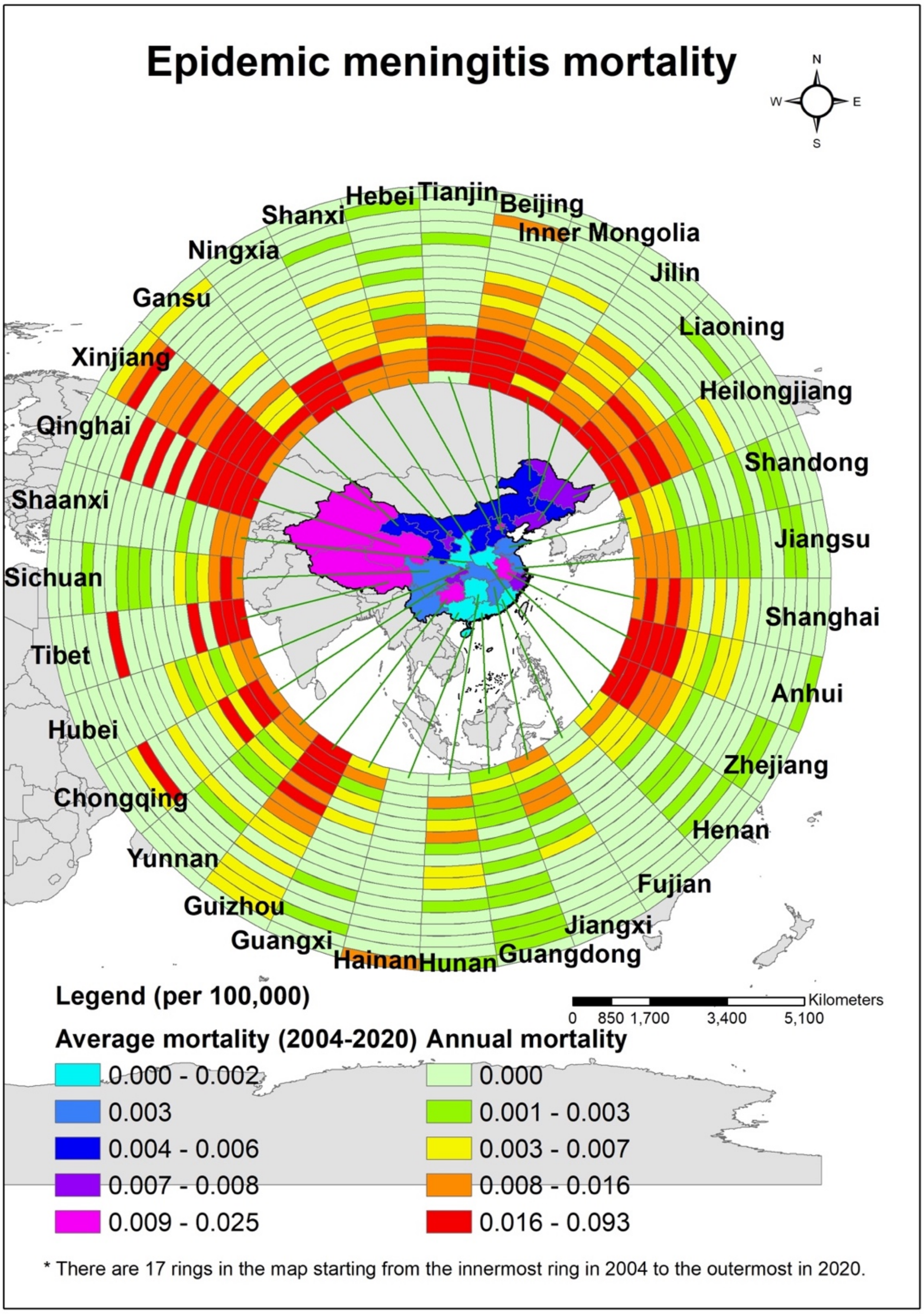

Supplementary Figure 2. Spatiotemporal distribution of the mortality due to Epidemic Cerebrospinal Meningitis in 31 province-level units, from 2004 to 2020. The spatiotemporal clusters of **Epidemic Cerebrospinal Meningitis**. All the 17 years' mortality data for 31 provinces were used, with a maximum cluster population size of 10% to minimize false clusters, and a maximum temporal window of three years to examine the clusters. The local risk ring maps were also done by the ArcGIS software. The 14 rings contain data for each year studied, with the innermost ring bearing data for 2004, and moving outwards through the years to the outermost ring bearing data for 2020.

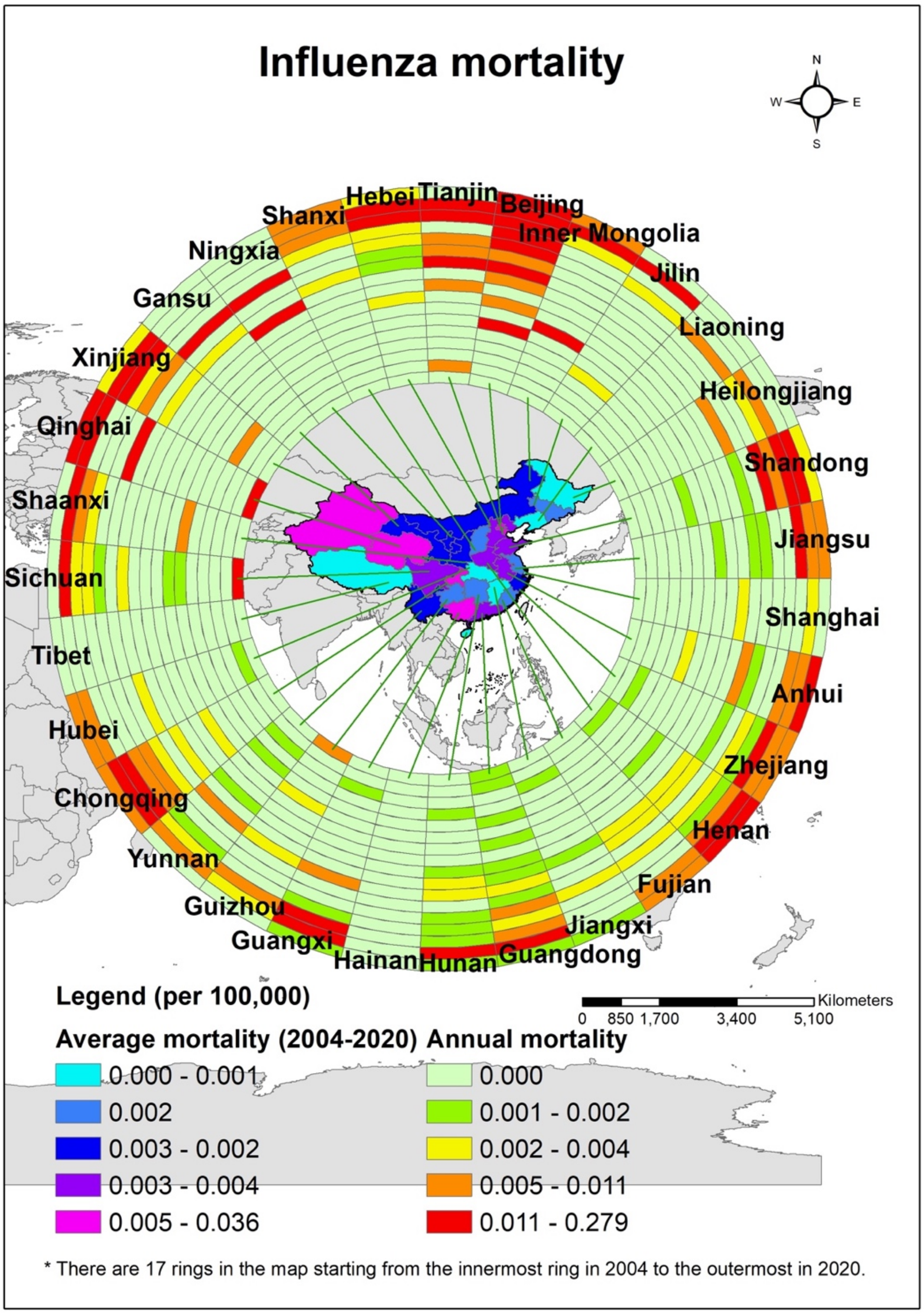

Supplementary Figure 3. Spatiotemporal distribution of the mortality due to seasonal influenza in 31 province-level units, from 2004 to 2020.

The spatiotemporal clusters of seasonal influenza. All the 17 years' mortality data for 31 provinces were used, with a maximum cluster population size of 10% to minimize false clusters, and a maximum temporal window of three years to examine the clusters. The local risk ring maps were also done by the ArcGIS software. The 14 rings contain data for each year studied, with the innermost ring bearing data for 2004, and moving outwards through the years to the outermost ring bearing data for 2020.

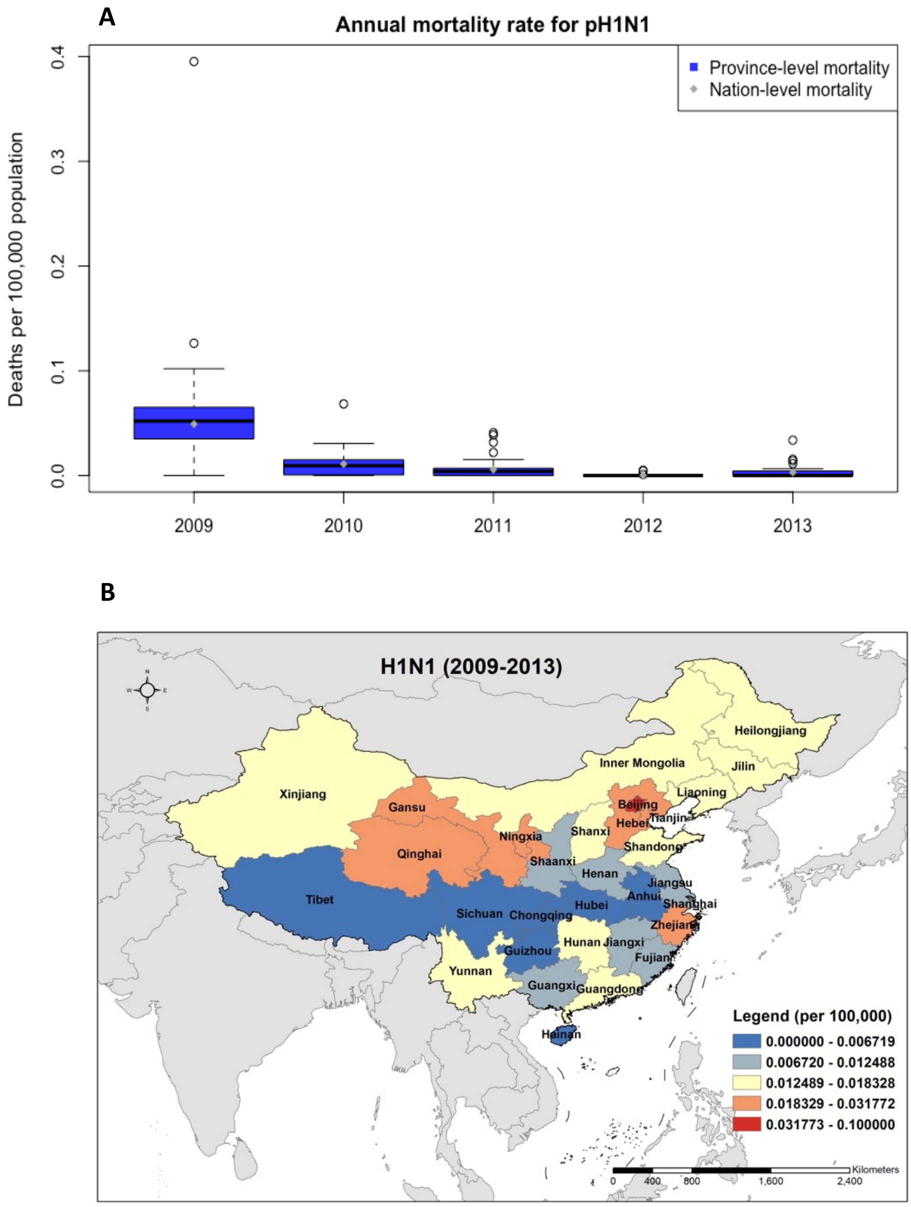

Supplementary Figure 4. Spatiotemporal distribution of the mortality due to pH1N1 reported in China, 2009-2013 in China. (A) The epidemic trend for the annual mortality rate from pH1N1 in overall China, 2009-2013; The bottom border of the boxes indicates the 25th percentile; middle line, the 50th percentile; and the top border indicates the 75th percentile across all 31 provinces; whiskers indicate the full range across all 31 provinces; and the dots indicate the national-level rate; **(B)** Choropleth maps of the average annual mortality of pH1N1, by region, based on the annual mortality per 100 000 people in China during 2009-2013.

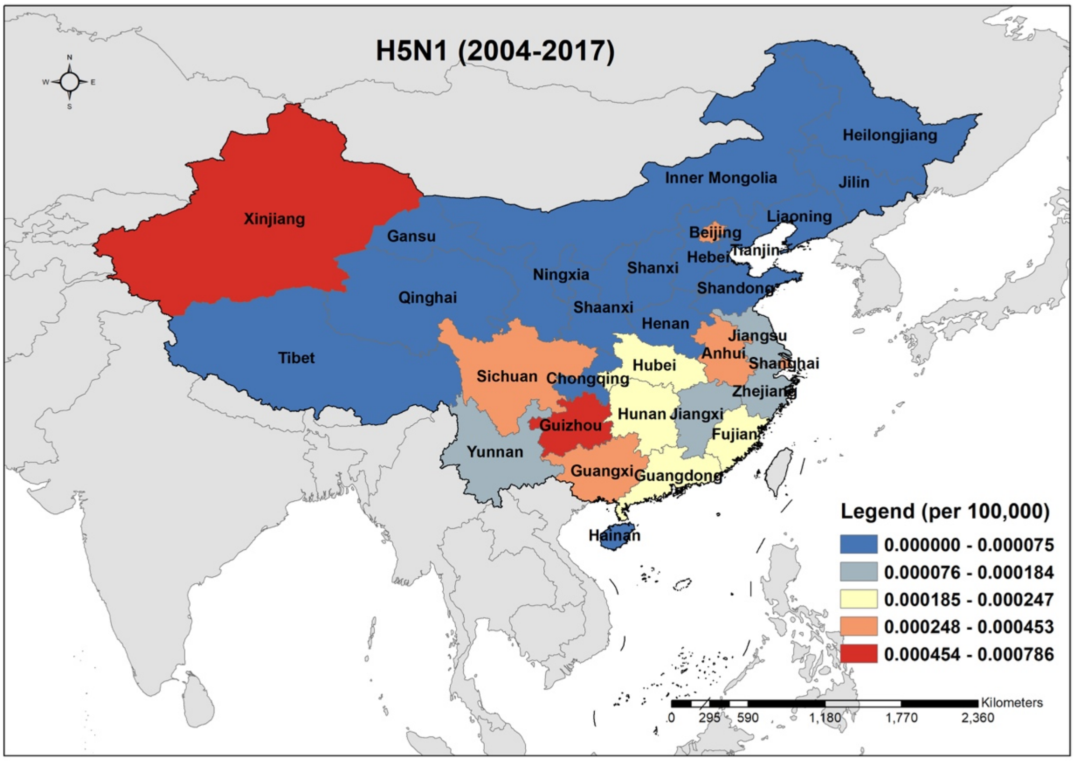

Supplementary Figure 5. The epidemic trend for the annual mortality rate from avian H5N1 influenza in China, 2004-2017. Choropleth maps of the average annual mortality of avian H5N1 influenza, by region, based on the annual mortality per 100 000 people.

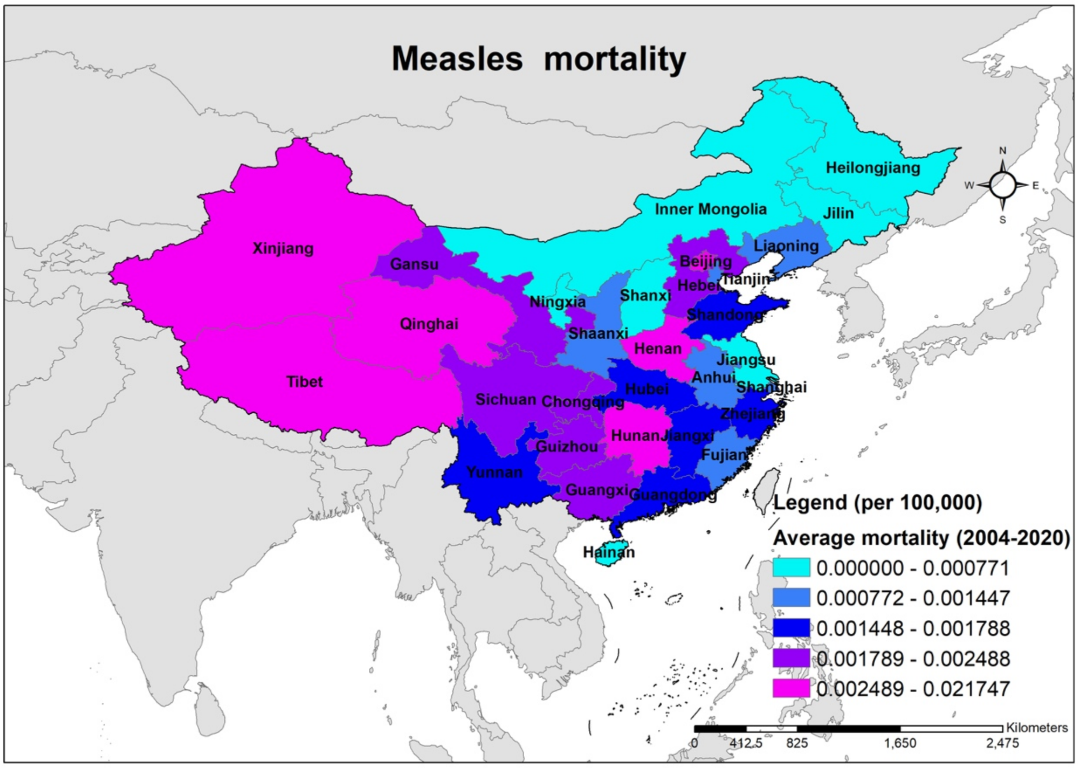

Supplementary Figure 6. The epidemic trend for the annual mortality rate from measles in China, 2004-2020. Choropleth maps of the average annual mortality of measles, by region, based on the annual mortality per 100 000 people in China during 2004-2020.

Supplementary Table 1. The classification of 40 notifiable infectious diseases reported from National Reporting System for Infectious Diseases in China since 2003

Classification	Name of notifiable infectious diseases
A	plague,<br>cholera
B	SARS (Severe acute respiratory syndrome),<br>HIV infection,<br>hepatitis (A, B, C, E),<br>poliomyelitis,<br>H5N1,<br>measles,<br>haemorrhagic fever,<br>rabies,<br>encephalitis B, dengue,<br>anthrax,<br>bacillary dysentery and amoebic dysentery,<br>tuberculosis,<br>typhoid and paratyphoid,<br>epidemic cerebrospinal meningitis,<br>pertussis,<br>diphtheria,<br>neonatal tetanus,<br>scarlet fever,

	brucellosis,<br>gonorrhoea, syphilis,<br>leptospirosis,<br>schistosomiasis,<br>malaria,<br>H7N9<br>COVID-19
C	seasonal influenza [(2009 pandemic influenza A(H1N1) was grouped into seasonal influenza after 2013]<br>mumps,<br>rubella,<br>acute haemorrhagic conjunctivitis,<br>leprosy,<br>typhus,<br>kala-azar,<br>hydatid disease,<br>filariasis,<br>HFMD (hand, foot, and mouth disease),<br>OID (infectious diarrhoeal diseases other than cholera, bacterial and amoebic dysentery, typhoid and paratyphoid).

Notes: (1) the 10 notifiable respiratory diseases in this research are highlighted in red color.

(2) National Reporting System for Infectious Diseases: In response to the 2003 severe acute respiratory syndrome (SARS) outbreak, the Chinese government established a web-based reporting system for selected infectious diseases. This system covers the Chinese population of 1.3 billion across 31 provinces in China. It included 40 notifiable infectious diseases characterized by wide prevalence or great harm, and classified them into classes A, B, and C, with severity decreasing across classes.

Supplementary Table 2: The diagnostic criteria for pulmonary tuberculosis in China

Standard Name	Diagnostic Criteria of Pulmonary Tuberculosis
**1 Scope**	This standard is applicable to the diagnosis of tuberculosis in all kinds of medical and health institutions and their medical personnel at all levels in the country.
**2 Terms and definitions**	2.1 Pulmonary tuberculosis<br>2.2 Mycobacterium tuberculosis
**3 Diagnostic basis**	
**3.1 Epidemiologic Linkage**	The patient has an epidemiologic history of contact with acute active pulmonary tuberculosis
**3.2 Clinical manifestations**	3.2.1 Symptoms: Cough, sputum or blood or haemoptysis in sputum lasting longer than two weeks
	3.2.2 Physical examination: A physical examination is done to assess the patient's general health. It cannot be used to confirm or rule out TB. However, certain findings are suggestive of TB; for example, blood in the sputum, significant weight loss and drenching wet rales, wheezing sound, superficial lymphadenopathy, and night sweats may be due to TB.
**3.3 Chest Imaging Examination**	3.3.1 Primary pulmonary tuberculosis: Primary pulmonary lesions and intrathoracic lymphadenopathy, or simple intrathoracic lymphadenopathy;<br>3.3.2 Haematogenous disseminated pulmonary tuberculosis: Miliary shadow with uniform size and density in both lungs;

	3.3.3 Secondary tuberculosis: The chest imaging manifestations of secondary pulmonary tuberculosis are diverse. In light cases, patches, nodules and striations are the main manifestations, or tuberculoma or solitary voids; in heavy cases, lobar infiltration, caseous pneumonia, multiple void formation and bronchial dissemination are the main manifestations; in repeated delays of progression, pulmonary lesions may occur, the volume of damaged lung tissue is reduced, multiple thick-walled fibrous holes, secondary bronchiectasis, or multiple calcification are the main manifestations. Pulmonary hilum and mediastinum structure show traction displacement, thoracic collapse, pleural thickening and adhesion; other lung tissues show compensatory emphysema and new and old bronchial disseminated lesions;<br>3.3.4 Tuberculosis of trachea and bronchi: The main manifestations of tracheobronchial tuberculosis are irregular thickening of the trachea or bronchial wall, stenosis or obstruction of the lumen, secondary atelectasis or consolidation, bronchiectasis, and other bronchial disseminated lesions in the distal lung tissue of the stenosed bronchus;<br>3.3.5 Tuberculous Pleurisy: Tuberculous pleurisy is divided into dry pleurisy and exudative pleurisy. Dry pleurisy is an early inflammatory response of the pleura, usually without obvious imaging manifestations; exudative pleurisy is mainly manifested as pleural effusion; and pleural effusion can be manifested as a small or medium amount of free effusion, or a limited effusion in any part of the pleura. Those patients with slow absorption are often accompanied by pleural thickening and adhesion, and can also evolve into pleural tuberculoma and empyema.
**3.4 Laboratory Tests**	3.4.1 Bacteriological test: (a) Positive for *Mycobacterium tuberculosis* by smear microscopy; (b) Mycobacterium was positive in culture and identified as *Mycobacterium tuberculosis* complex.<br>3.4.2 Molecular Biology Examination: Positive nucleic acid for *Mycobacterium tuberculosis*<br>3.4.3 Pathological examination of tuberculosis: Histopathological changes typical of tuberculosis<br>3.4.4 Immunological examination<br>3.4.4.1 Moderate or strong positive tuberculin skin test;<br>3.4.4.2 Positive IFN-gamma release test;<br>3.4.4.3 Positive *Mycobacterium tuberculosis* antibody test.

	3.4.5 Bronchoscopy: Bronchoscopy can directly reveal the pathological changes in the trachea and bronchus, as well as allow collection of aspirate secretions, brush samples and biopsies.
**4. Diagnostic principles**	The diagnosis of pulmonary tuberculosis is based on etiology (including bacteriology and molecular biology), combined with epidemiological history, clinical manifestations, chest imaging, related auxiliary examinations and differential diagnosis; together, these provide a comprehensive diagnosis. Pathogenic and pathological results are used as the basis for confirmation. In the diagnosis of pulmonary tuberculosis in children, besides a sputum etiology examination, young patients should also undergo a gastric etiology examination.
**5. Diagnosis and Classification**	
**5.1 Suspected cases**	Those who meet one of the following criteria:<br>(a) having any of the 3.3 items;<br>(b) Children under 5 years old with 3.2 and 3.1, 3.4.4.1 and 3.4.4.2.
**5.2 Clinically diagnosed cases**	Other pulmonary diseases are excluded by differential diagnosis, and one of the following criteria is met:<br>(a) with any of 3.3 and 3.2;<br>(b) with any of 3.3 and 3.4.4.1;<br>(c) with any of 3.3 and 3.4.4.2;<br>(d) with any of 3.3 and 3.4.4.3;<br>(e) with any of the 3.3 items and with extrapulmonary pathological examination confirming tuberculosis lesions;<br>(f) Tracheal and bronchial tuberculosis are diagnosed with 3.3.4 and 3.5.
**5.3 Confirmed cases**	5.3.1 Diagnosis of sputum smear positive tuberculosis<br>Those who meet one of the following criteria:<br>(a) 2 sputum smears with acid-fast bacilli conform to 3.4.1.a;<br>(b) The acid-fast bacillus test of one sputum specimen conforms to 3.4.1.a, with any of the 3.3 items concomitantly;<br>(c) The acid-fast bacilli test of one sputum specimen is 3.4.1.a, and the culture of *Mycobacterium* in one sputum specimen is 3.4.1.b.

	5.3.2 Diagnosis of *Mycobacterium* tuberculosis<br>In accordance with any of 3.3, at least 2 sputum smears are negative, and mycobacterium culture agrees with 3.4.1.b.<br>5.3.3 Molecular Biology Examination Positive Diagnosis of Tuberculosis<br>Compliance with either 3.3 or 3.4.2.<br>5.3.4 Diagnosis of Pulmonary Tuberculosis Positive by Histopathological Examination of Lung<br>Compliance with 3.4.3.<br>5.3.5 Diagnosis of Tracheobronchial Tuberculosis<br>Those who meet one of the following criteria:<br>(a) Those with 3.5 and 3.4.3 coincidence in pathological examination of trachea and bronchus;<br>(b) Those with 3.5 and 3.4.1.a or 3.4.1.b or 3.4.2 of tracheal and bronchial secretions.<br>5.3.6 Diagnosis of tuberculous pleurisy<br>Those who meet one of the following criteria:<br>(a) Those with 3.3 pleural effusion or pleural pathology accorded with 3.4.3;<br>(b) Those with 3.3 and 3.4.1.a or 3.4.1.b or 3.4.2 of pleural effusion etiology.
**Chinese Standard Number**	WS 288-2017
**Issued by**	National Health Commission of the People’s Republic of China
**Date issued**	November 9, 2017
**Official Source**	http://www.nhc.gov.cn/ewebeditor/uploadfile/2017/11/20171128164254246.pdf

Supplementary Table 3: The diagnostic criteria for diagnosis for Epidemic Cerebrospinal Meningitis in China

Standard Name	Diagnostic Criteria for Diagnosis of Epidemic Cerebrospinal Meningitis
**1 Scope**	This standard is applicable to the diagnosis of epidemic cerebrospinal meningitis by medical and health institutions at all levels and their medical personnel.
**2 Terms and definitions**	CFU/mL:colony-forming unit/mL<br>DIC: disseminated or diffuse intravascular coagulation<br>DNA: deoxyribonucleic acid<br>ELISA: enzyme-linked immunosorbent assay<br>IgG: immunoglobulin G<br>OD: optical density<br>PBS: phosphate buffer saline<br>Real-time PCR: real-time polymerase chain reaction<br>SBA: serum bactericidal assays<br>TTC: triphenyl tetrazolium chloride<br>RPM:revolutions per minute<br>WHO:World Health Organisation
**3 Diagnostic basis**	
**3.1 Epidemiologic Linkage**	The disease occurs or prevails locally, or the patient has a history of residence or travel in epidemic areas within 10 days before the onset of epidemic cerebrospinal meningitis.

**3.2 Clinical manifestations**	3.2.1 Incubation period: Hours to 10 days, usually 2 to 3 days.<br>3.2.2 Main clinical symptoms and signs<br>3.2.2.1 Fever, headache, vomiting, and/or meningeal irritation. Anterior fontanel eminence can be seen in infants and young children. Severe patients may have varying degrees of consciousness disorder and/or infectious toxic shock.<br>3.2.2.2 The skin and mucosa shows ecchymosis (spots). Stasis can rapidly expand and fuse into patches.
**3.3 Laboratory Testing**	3.3.1 Blood routine: Total white blood cell count and neutrophil count are significantly increased.<br>3.3.2 Cerebrospinal Fluid Routine: Typical changes are increased pressure, appearance of turbid rice soup or pus; white blood cell count is significantly increased, mainly polymorphonuclear white blood cells are increased; sugar and chloride are significantly decreased, protein content is increased. In the early stage of the course, only the pressure is increased, the appearance is clear, and then typical changes occur. The CSF of patients with fulminant shock type is usually clear, and no changes occur in protein, cell number and sugar.<br>3.3.3 Etiology<br>3.3.3.1 Gram-negative Nephrobacteriaceae can be found in polymorphonuclear leukocytes or extracellularly by smear examination of blood stasis (spot) tissue fluid and cerebrospinal fluid.<br>3.3.3.2 Cerebrospinal fluid, blood and blood stasis tissue fluid are positive for *Neisseria meningitidis*.<br>3.3.3.3 Cerebrospinal fluid, blood and blood stasis (spot) tissue fluid are positive for specific nucleic acids of *Neisseria meningitidis*.<br>3.3.4 Immunology<br>3.3.4.1 Cerebrospinal fluid samples of the acute phase are positive for *Neisseria meningitidis* specific polysaccharide antigen.<br>3.3.4.2 The titre of specific IgG antibody against *Neisseria meningitidis* is 4 or more times higher in convalescent serum than in acute serum.

**4. Diagnostic principles**	Diagnosis of suspected cases and/or clinically diagnosed cases is made according to the epidemiological history and clinical manifestations, routine blood test results and/or routine cerebrospinal fluid test results. Diagnosis requires etiological or immunological test results for *Neisseria meningitides*, and further etiological cluster diagnosis is made for cases with a positive etiological test.
**5. Diagnosis and Classification**	
**5.1 Suspected cases**	Simultaneous agreement with 3.1 and 3.2.2.1, and with either 3.3.1 or 3.3.2.
**5.2 Clinically diagnosed cases**	Simultaneous agreement with 5.1 and 3.2.2.2.
**5.3 Confirmed cases**	Simultaneous conforming to 5.1 or 5.2 and to either 3.3.3 or 3.3.4.
**Chinese Standard Number**	WS 295-2019
**Issued by**	National Health Commission of the People's Republic of China
**Date issued**	January 2, 2019
**Official Source**	http://www.nhc.gov.cn/fzs/s7852d/201901/9493bdd1549b4908be18beb6007b009d/files/5353d61afc41476e90856896e34fea16.pdf

Supplementary Table 4: The diagnostic criteria for 2009 pandemic H1N1 influenza in China

Standard Name	Diagnostic Criteria of 2009 Pandemic H1N1 Influenza
**1 Scope**	This standard is applicable to the diagnosis of **2009 pandemic H1N1 Influenza** in all kinds of medical and health institutions and their medical personnel at all levels in the country.
**2 Terms and definitions**	**2009 pandemic H1N1 Influenza**
**3 Diagnostic basis**	
**3.1 Epidemiologic Linkage**	There is close contact with confirmed cases of influenza A (H1N1) within 7 days before onset, and flu-like clinical manifestations appeared.
**3.2 Clinical manifestations**	Usually manifests as influenza-like symptoms, including fever, sore throat, runny nose, stuffy nose, cough, sputum, headache, general soreness and fatigue. Some cases have vomiting and/or diarrhoea. A few cases have mild upper respiratory symptoms and no fever. Signs include pharyngeal congestion and tonsillar enlargement.<br>Complications such as pneumonia may occur. A few cases progress rapidly with respiratory failure and multiple organ dysfunction or failure.<br>Influenza-like symptoms in newborns and infants are often atypical, including low fever, lethargy, feeding difficulties, shortness of breath, apnea, cyanosis and dehydration. Children are prone to wheezing, and some children suffer from central nervous system damage.<br>Women infected with influenza A (H1N1) in the second and third trimesters of pregnancy often show shortness of breath and are prone to pneumonia and respiratory failure. Infection of pregnant women with influenza A (H1N1) may lead to abortion, premature delivery, foetal distress, intrauterine foetal death and other adverse pregnancy outcomes.<br>Infection can aggravate the original basic diseases and present corresponding clinical manifestations.<br>Severe illness can lead to death.

**3.3 Chest Imaging Examination**	The basic imaging features of influenza A (H1N1) pneumonia on chest X-ray and CT are pulmonary patchy shadows and consolidation or ground glass density, which can be combined with reticular, linear and small nodule shadows. Patchy shadows are localised or multiple, with diffuse distribution, and are mostly bilateral lesions. Pleural effusion may be combined. In children, the pulmonary patchy shadows appear earlier, more frequently and more scattered and were prone to hyperinflation. The imaging manifestations can change rapidly. When the disease progresses, the lesions expand and fuse. Pneumothorax and mediastinal emphysema can appear.
**3.4 Laboratory Tests**	1. Peripheral haemogram: The total number of white blood cells is normal or decreased. Some children with severe illness may have an increase in the total number of white blood cells.<br>2. Blood biochemical examination: hypokalaemia occurs in some cases, while creatine kinase, aspartate aminotransferase, alanine aminotransferase and lactate dehydrogenase increase in some cases.<br>3. Pathogenic examination:<br>(1) Viral nucleic acid detection: RT-PCR (preferably real-time RT-PCR) is used to detect influenza A H1N1 virus nucleic acid in respiratory tract samples (pharynx swab, nasal swab, nasopharyngeal or tracheal extracts, sputum).<br>(2) Virus isolation: A H1N1 influenza virus can be isolated from respiratory specimens.<br>(3) Serum antibody test: Dynamic detection of specific antibody levels of influenza A H1N1 virus in two sera shows increases of 4 or more times.
**4. Diagnostic principles**	Diagnosis is mainly combined with epidemiological history, clinical manifestations and etiological examination. Early detection and diagnosis are the key to prevention, control and effective treatment.
**5. Diagnosis and Classification**	
**5.1 Suspected cases**	5.1 Close contact with confirmed cases of influenza A (H1N1) in the infectious period and influenza-like clinical manifestations occurring within 7 days before the onset of the disease.<br>Close contact includes the diagnosis, treatment and care of patients with influenza A (H1N1) during the infectious period without effective protection; living with patients; coming in contact with patients' respiratory secretions, body fluids, etc.<br>5.2 Influenza-like clinical manifestations, positive test for influenza A virus, no further detection of virus subtypes.

**5.2 Clinically diagnosed cases**	Clinical diagnosis is limited to the following: in the same outbreak of influenza A (H1N1), cases with flu-like symptoms that have not been laboratory confirmed can be diagnosed clinically when other diseases causing flu-like symptoms are excluded. An outbreak of influenza A (H1N1) refers to an abnormal increase in influenza-like cases in a region or unit in a short time. The outbreak of influenza A (H1N1) is confirmed by laboratory tests.
**5.3 Confirmed cases**	Influenza-like clinical manifestations occur, with one or more of the following laboratory results:<br>5.3.1 The test for nucleic acid of influenza A (H1N1) virus is positive (real-time RT-PCR and RT-PCR could be used).<br>5.3.2 Isolation of influenza A H1N1 virus;<br>5.3.3 The specific antibody level of influenza A (H1N1) virus in two sera is increased by four or more times.
**Chinese Standard Number**	2010 version
**Issued by**	National Health Commission of the People's Republic of China
**Date issued**	September 3, 2010
**Official Source**	http://www.nhc.gov.cn/xxgk/pages/viewdocument.jsp?dispatchDate=&staticUrl=/zwgkzt/wsbysj/201005/47250.shtml&wenhao=无&utitle=卫生部办公厅关于印发《甲型 H1N1 流感诊疗方案（2010 年版）》的通知&topictype=&topic=&publishedOrg=医政司&indexNum=000013610/2010-03343&manuscriptId=47250

Supplementary Table 5: The diagnostic criteria for influenza in China

Standard Name	Diagnostic Criteria of Influenza
**1 Scope**	This standard is applicable to all levels of medical and health institutions and personnel in the country for the diagnosis and reporting of influenza.
**2 Terms and definitions**	Type of influenza virus; Subtype of influenza A virus; Influenza-like illness
**3 Diagnostic basis**	
**3.1 Epidemiologic Linkage**	In the local epidemic season (i.e. winter and spring in northern China and winter, spring and summer in southern China), a large number of upper respiratory tract infections occur in a unit or area, or the number of patients with upper respiratory tract infections in hospital outpatient and emergency departments increases significantly.
**3.2 Clinical manifestations**	3.2.1 The common manifestations are acute fever (axillary body temperature (> 38 C), chills, headache, dizziness, soreness, fatigue and other toxic symptoms, as well as respiratory symptoms, such as pharyngalgia and dry cough, but the catarrhal symptoms are often not obvious.<br>3.2.2 A few cases can have anorexia accompanied by gastrointestinal symptoms, such as abdominal pain, abdominal distention, vomiting and diarrhoea.<br>3.2.3 A few cases can also be complicated with sinusitis, otitis media, laryngitis, bronchitis, pneumonia, and even respiratory and circulatory failure and death.<br>3.2.4 Children under two years of age, or those with chronic underlying diseases, may have lower respiratory sounds and wet beeps or wheezing sounds in both lungs, but no signs of lung consolidation.<br>3.2.5 Thoracic X-ray examination of severe patients can show unilateral or bilateral pulmonary parenchymal lesions, and a few lesions may be accompanied by pleural effusion.

	3.2.6 The total number of white blood cells in peripheral haemogram is neither high nor low, and the lymphocytes increase relatively. The total numbers of white blood cells and lymphocyte decrease in severe patients.
**3.3 Laboratory Tests**	The procedures for collecting, transporting and treating influenza specimens can be found in Appendix G.<br>3.3.1 Influenza viruses are isolated and identified from patients' respiratory tract specimens (see Appendix A).<br>3.3.2 The titre of serum anti-influenza virus antibody is 4 or more times higher in convalescent patients than in acute patients (see Appendix B and C).<br>3.3.3 Influenza virus specific nucleic acid (see Appendix D) or specific antigen (see Appendix E) is detected in the patients' respiratory tract specimens.<br>3.3.4 After the virus proliferates in sensitive cells for one generation, the specific nucleic acid of influenza virus gives a positive test (see Appendix D for details) or a specific antigen is detected (see Appendix E).
**4. Diagnostic principles**	If only clinical manifestations in a non-epidemic season are considered, distinguishing influenza virus from other pathogens is difficult, especially for diseases caused by other respiratory pathogens. The diagnosis of influenza cases often requires laboratory diagnosis. However, in the flu season, when a large number of patients with upper respiratory tract infections or outpatient and emergency upper respiratory tract infections in a local unit or local area increase significantly, the patients with corresponding clinical manifestations can be considered clinically diagnostic cases of influenza. See Appendix F for the etiology, epidemiology and clinical manifestations of influenza.
**5. Diagnosis and Classification**	
**5.1 Clinically diagnosed cases**	Those with any of the clinical manifestations in 3.1 and 3.2.

**5.2 Confirmed cases**	5.2.1 Influenza-like cases with any of 3.3.<br>5.2.2 Clinically diagnosed cases with any of 3.3.
**Chinese Standard Number**	WS285-2008
**Issued by**	National Health Commission of the People's Republic of China
**Date issued**	February 28, 2008
**Official Source**	

Supplementary Table 6: The diagnostic criteria for human infection with avian influenza virus in China

Standard Name	Diagnostic Criteria of Human Infection with Avian Influenza Virus
**1 Scope**	This standard is applicable to the diagnosis and reporting of human infection with avian influenza virus in medical institutions and their staff at all levels throughout the country.
**2 Terms and definitions**	Human-avian influenza, Highly pathogenic avian influenza (HPAI), Influenza-like illness
**3 Diagnostic basis**	
**3.1 Epidemiologic Linkage**	3. 1. Within 7 days before the onset of the disease, patients are exposed to poultry, especially sick and dead poultry (including wild and poultry), or their excreta, secretions and eggs laid within 7 days, or exposed to the environment polluted by poultry excreta and secretions.<br>3.1.2 Within 14 days before the onset of the disease, patients visited live poultry trading and slaughtering markets.<br>3.1.3 Within 14 days before the onset of the disease, close contacts were made with suspected cases of human avian influenza, IgG diagnosis or laboratory confirmed cases, including living together or nursing cases.<br>3.1.4 Within 14 days before the onset of the disease, the patient visited an area where 3 dead poultry were found.<br>3.1.5 High-risk occupational history: Laboratory personnel engaged in raising , selling, slaughtering, processing poultry or diagnosing #poultry workers may be exposed to animal and human avian influenza viruses or potentially infectious materials; personnel who do not take strict personal protective measures to deal with animals during highly pathogenic avian influenza outbreaks; personnel who do not take strict personal protective measures to diagnose, treat and care for impending or suspected human infections; personnel involved in medical care of confirmed cases in laboratory personnel.

**3.2 Clinical manifestations**	(a) The incubation period is usually 1–7 days, usually 2–4 days. The patient presents with acute onset and similar early manifestations. Human avian influenza A: Mainly fever, the body temperature mostly stays above 39 degrees Celsius; fever can be accompanied by runny nose, nasal obstruction, cough, etc. Pharyngeal pain, headache, muscle soreness and general discomfort occur. Some patients may have nausea, abdominal pain, diarrhoea and watery stool. Isolated digestive tract symptoms can occur. (b) The condition of severe patients develops rapidly. Almost all patients have pneumonia with obvious clinical manifestations, which can lead to emergencies. Lung injury, acute respiratory distress syndrome (ARDS), pulmonary haemorrhage, pleural effusion, pancytopenia, multiple organ failure, shock and Reye syndrome symptoms can be complications. Secondary bacterial infection and septicaemia may occur. (c) The total number of white blood cells in peripheral blood is normal or decreased. The number of leukocytes decreases and enlarged lymph nodes increases in severe patients.s. (d) Signs: Severe patients may have pulmonary consolidation signs, etc.
**3.3 Chest Imaging Examination**	Patchy, multipatchy and fused unilateral or bilateral lesions appear at the beginning of the disease. Other occurrences are lateral lung consolidation, light shadows of pulmonary parenchyma exudation, flocculent ground glass density and rapid progress of severe patients, with pathological changes. The lesions show diffuse distribution in both lungs, without obvious segmental or lobar characteristics. A considerable number of cases developed into "white lung-like" changes, which could be combined with pleural effusion.
**3.4 Laboratory Tests**	3.4.1 Isolation of virus<br>Virus isolation is positive and confirmed by subtype identification.<br>3.4.2 Serological examination<br>3.4.2.1 Haemagglutination inhibition (HI) tests are performed in convalescent serum from the patients.<br>3.4.2.2 Microneutralisation test (MNT) and Avian Influenza Virus (HA) (H5 or H7 or H9 subtypes) antibody positive tests (HI antibody or neutralising antibody titre >80, excluding persons over 55 years old).<br>3.4.2.3 The titre of the serum antibody is 4 times higher in the convalescent stage than in the acute stage.<br>3.3.3 Virus Antigen and Nucleic Acid Detection<br>Human avian influenza specific nucleic acids, or specific H subtype antigens, are examined in clinical specimens.

**4. Diagnostic principles**	The diagnosis of human avian influenza cases needs to be combined with the epidemiological history, clinical manifestations and laboratory tests. Epidemiological history is an important, but not necessary, condition for diagnosis. Confirmed cases require stringent virological or serological evidence, especially evidence that serum antibody titres are four or more times higher in the convalescent stage than in the acute stage. For early and timely detection of human avian influenza cases, medical staff should inquire in detail about the epidemiological history of patients and make a diagnosis of suspected cases of human avian influenza according to the epidemiological history and clinical manifestations.
**5. Diagnosis and Classification**	
**5.1 Suspected cases**	The suspected cases of human avian influenza have any of the 3.1 items and no other cases of pneumonia with definite diagnosis.
**5.2 Clinically diagnosed cases**	Clinically diagnosed cases of human avian influenza include any of the following:<br>5.2.1 Has any item in 3.1 plus any item in 3.2 and conforms to any item in 3.3.2.1 and 3.3.2.2.<br>5.2.2 Diagnosis of suspected cases of human avian influenza is made when no further clinical specimens for laboratory confirmation are available, while others with a history of exposure are diagnosed as confirmed cases of human avian influenza if no basis exists for the diagnosis of other diseases.
**5.3 Confirmed cases**	Any of the following:<br>5.3.1 3.2 plus 3.3.1.<br>5.3.2 3.2 plus 3.3.2.<br>5.3.3 3.2 plus 3.3.3 and has been confirmed by two different laboratories.
**Chinese Standard Number**	WS284-2008
**Issued by**	National Health Commission of the People’s Republic of China

**Date issued**	February 28, 2008
**Official Source**	http://www.nhc.gov.cn/wjw/s9491/200802/39042/files/c12351d9bfec4aa1995d661811372fc0.pdf

Supplementary Table 7: The diagnostic criteria for measles in China

Standard Name	Diagnostic Criteria of Measles
**1 Scope**	This standard is applicable to the diagnosis of measles by medical and health institutions at all levels and their medical personnel
**2 Terms and definitions**	CPE ：cytopathic effect<br>DEPC：diethyl pyrocarbonate<br>ELISA ：enzyme-linked immunosorbent assay<br>IgG：immunoglobulin G<br>IgM：immunoglobulin M<br>RF：rheumatoid factor<br>RNA：ribonucleic acid<br>RT-PCR：reverse transcription-polymerase chain reaction<br>RPM：revolutions per minute<br>OD：optical density value<br>VTM：virus transportation medium
**3 Diagnostic basis**	
**3.1 Epidemiologic Linkage**	3.1.1 At 7-21 days before the onset of measles, a patient with measles had contact history.<br>3.1.2 The patient had a history of residence or travel in measles-endemic areas 7 to 21 days before the onset of measles.

**3.2 Clinical manifestations**	3.2.1 Fever, body temperature generally (> 38 C).<br>3.2.2 Red macular papules appear on the 3rd to 4th day of the course of the disease, and the skin between the rashes is normal. The order of eruption usually starts from the back of the ear and the face and extends from top to bottom to the whole body; it can involve the mucosa. The eruption lasts for 3 to 5 days.<br>3.2.3 Upper respiratory catarrhal symptoms, such as cough, runny nose and sneezing, as well as photophobia, tearing and conjunctivitis.<br>3.2.4 Measles mucosal plaques (Koplik plaques) are found on the oral and buccal mucosa at the early stage of onset (usually on the 2nd to 3rd day of the course).
**3.3 Laboratory Tests**	3.3.1 No live attenuated measles vaccine containing measles components were inoculated within 8 to 56 days before blood collection, but measles IgM is positive in blood samples within 28 days after measles emergence<br>3.3.2 Measles virus nucleic acid is positive in throat swabs or urine samples or is isolated from measles virus<br>3.3.3 The titre of measles IgG antibody increases more than 4 times in convalescent blood samples than in acute stage, or the antibody test is negative in the acute stage and positive in the convalescent stage.
**4. Diagnostic principles**	Diagnosis is made according to epidemiological history, clinical manifestations and laboratory results.
**5. Diagnosis and Classification**	
**5.1 Suspected cases**	The patient has 3.2.1, 3.2.2 and 3.2.3.
**5.2 Clinically diagnosed cases**	A suspected case meets any of the following criteria:<br>(a) Has 3.1.1 and/or 3.1.2, and has not been clearly diagnosed as other diseases;<br>(b) 3.2.4;<br>(c) No specimens were collected for laboratory testing, but no other diseases were definitely diagnosed.
**5.3 Confirmed cases**	The suspected cases had any one of 3.3.1, 3.3.2 and 3.3.3.

**Chinese Standard Number**	WS 296-2017
**Issued by**	National Health Commission of the People's Republic of China
**Date issued**	July 24, 2017
**Official Source**	http://www.nhc.gov.cn/ewebeditor/uploadfile/2017/07/20170727145913239.pdf

Supplementary Table 8: The diagnostic criteria for mumps in China

Standard Name	Diagnostic Criteria of Mumps
**1 Scope**	This standard is applicable to the diagnosis and reporting of mumps in all kinds of medical and health institutions and their staff at all levels in China.
**2 Diagnostic basis**	
**2.1 Epidemiologic Linkage**	The patient has a history of contact with patients with mumps or an epidemic of mumps has occurred in the area from 14 to 28 days before the onset of the disease.
**2.2 Clinical manifestations**	2.2.1 Fever, headache, fatigue, loss of appetite, etc.<br>2.2. Swelling and pain of the unilateral or bilateral parotid glands and/or other salivary glands are aggravated when opening the mouth, chewing or eating acidic foods.<br>2.2.3 Headache, vomiting, meningeal irritation or changes of consciousness occur in patients with meningoencephalitis.<br>2.2.4 When accompanied by orchitis, swelling and pain occurs in the testis or epididymis.<br>2.2.5 Patients with pancreatitis show vomiting, upper and middle abdominal pain and tenderness.
**2.3 Laboratory Tests**	2.3.1 Leukocyte counts and routine urine tests are normal; leucocyte counts can be increased in patients with orchitis.<br>2.3.2 Most (90%) patients have increased serum and urinary amylase in the early stage of onset. In patients with meningoencephalitis without parotid gland enlargement, amylase can also be elevated in the blood and urine. The increase in serum lipase is helpful in the diagnosis of pancreatitis.<br>2.3.3 About half of the patients may have cerebrospinal fluid changes in viral meningoencephalitis.

	2. 3. 4 The mumps virus specific IgM antibody is detected in the serum of patients who had not been vaccinated with live attenuated vaccine in the past month.<br>2.3.5 The titre ratio of mumps virus IgG antibody in convalescent and acute sera (interval 2 to 4 weeks) is 4 or more times higher (including positive antibody conversion).<br>2.3.6 Mumps virus is isolated from saliva, urine, cerebrospinal fluid and other body fluids (Appendix A.2 Mumps virus can be isolated by any method).
**3. Diagnostic principles**	The diagnosis of parotid gland enlargement depends mainly on the epidemiological history and parotid gland and/or other acute salivary gland enlargement. Laboratory-specific examinations are required for confirmed cases.
**4. Diagnosis and Classification**	
**4.1 Suspected cases**	Any of the following is a suspected case:<br>4.1.1 Compliance with 2.2.2;<br>4.1.2: 2.1 and 2.2.1;<br>4.1.3 Agrees with 2.1 and 2.2.3;<br>4.1.4 Agrees with 2.1 and 2.2.4;<br>4.1.5 Agrees with 2.1 and 2.2.5.
**4.2 Clinically diagnosed cases**	Any of the following is a clinically diagnosed case:<br>4.2.1 Compliance with 2.2.2 and 2.2.1;<br>4.2.2 Compliance with 2.2.2 and 2.2.3;<br>4.2.3 Compliance with 2.2.2 and 2.2.4;<br>4.2.4 Compliance with 2.2.2 and 2.2.5;<br>4.2.5 Compliance with 2.1 and 2.2.1 and 2.3.1;<br>4.2.6 Compliance with 2.1 and 2.2.1 and 2.3.2;<br>4.2.7 Is consistent with 2.1 and 2.2.1 and 2.3.3.

**4.3 Confirmed cases**	In accordance with any of the following items,<br>5.3.1 Suspected cases or clinically diagnosed cases with 2.3.4<br>5.3.2 Suspected cases or clinically diagnosed cases with 2.3.5<br>5.3.3 suspected cases or clinically diagnosed cases with 2.3.6
**Chinese Standard Number**	WS 270-2007
**Issued by**	National Health Commission of the People's Republic of China
**Date issued**	April 17, 2007
**Official Source**	http://www.nhc.gov.cn/wjw/s9491/200704/38797/files/4b993dfd62834ccda16b8240ab078a00.pdf

Supplementary Table 9: The diagnostic criteria for rubella in China

Standard Name	Diagnostic Criteria of Rubella
**1 Scope**	This standard is applicable to the diagnosis and report of rubella and congenital rubella syndrome in all medical and health institutions and their staff at all levels in China.
**2 Terms and definitions**	RNA: ribonucleic acid<br>CRS: congenital rubella syndrome<br>IgM: immunoglobulin M<br>IgG: immunoglobulin G<br>CPE: cytopathic effect<br>RT: reverse transcription<br>PCR: polymerase chain reaction<br>ELISA: enzyme-linked immunosorbent assay
**3 Diagnostic basis**	
**3.1 Rubella**	
**3.1.1 Epidemiologic Linkage**	No rubella has ever occurred before, and a clear contact history had occurred with confirmed rubella patients within 14 days and 21 days before the onset of the disease.

**3.1.2 Clinical manifestations**	3.1.2.1 Fever, generally low or moderate<br>3.1.2.2. Red congestive maculopapular rash over the whole skin within 2 days of onset.<br>3.1.2.3 Posterior auricular, occipital, cervical lymph node enlargement or conjunctivitis or joint pain (arthritis).
**3.1.3 Laboratory Tests**	3. 1. 3.1 Rubella virus is isolated from throat swabs or urine samples, or rubella virus nucleic acid is detected.<br>3.1.3.2 Serum is positive for rubella IgM antibody (no live attenuated rubella vaccine were inoculated in the past month)<br>3. 1. 3. 3. The titres of serum rubella IgG antibody or rubella haemagglutination inhibitory antibody are more than 4 times higher in the convalescent stage than in the acute stage.<br>3.1.3.4 The antibody test is negative in the acute phase and positive in the convalescent phase.
**4. Diagnostic principles**	Clinical diagnosis is made according to clinical manifestations and epidemiology. The diagnosis is confirmed according to the results of the serum rubella antibody test or the rubella etiology test.
**5. Diagnosis and Classification**	
**5.1 Rubella**	
**5.1.1 Suspected cases**	Compliance with either of the following:<br>5.1.1.1 3.1.2.1 and 3.1.2.2<br>5.1.1.2 3.1.2.2 and 3.1.2.3
**5.1.2 Clinically diagnosed cases**	Suspected cases and concurrent coincidence 3.1.1

**5.1.3 Confirmed cases**	Simultaneous merger of suspected cases with any of 3.1.3
**Chinese Standard Number**	WS297-2008
**Issued by**	National Health Commission of the People’s Republic of China
**Date issued**	December 11, 2008
**Official Source**	http://www.nhc.gov.cn/wjw/s9491/200908/42159/files/f6ed02c7a47a49f9a30570ce2adbf058.pdf

Supplementary Table 10: The diagnostic criteria for pertussis in China

Standard Name	Diagnostic Criteria of Pertussis
**1 Scope**	This standard is applicable to the diagnosis and reporting of pertussis by medical and health institutions and their staff at all levels in China.
**2 Terms and definitions**	
**3 Diagnostic basis**	
**3.1 Epidemiologic Linkage**	The incidence of pertussis occurs in spring and summer. Pertussis is epidemic in the area. The patient has a history of close contact with pertussis patients.<br>The patient has no history of vaccination.
**3.2 Clinical manifestations**	3.2.1 Typical cases have paroxysmal and spastic cough and persistent cough for more than 2 weeks.<br>3.2.2 Infants with atypical cases have recurrent apnea, asphyxia, cyanosis and bradycardia symptoms, or intermittent paroxysmal cough; adolescents and adults have atypical mild symptoms. Symptoms of catarrhal, spastic and convalescent stages are shortened or show no obvious stages other than a long-term cough lasting more than two weeks.
**3.3 Laboratory Tests**	3.3.1 Peripheral white blood cell count and lymphocyte count increase significantly.<br>3.3.2 Bordetella pertussis is isolated from sputum and nasopharyngeal secretions.<br>3.3.3 The serum specific antibody increases by more than 4 times in the convalescent stage than in the acute stage.
**4. Diagnostic principles**	Pertussis cases can be diagnosed based on the epidemiological history, clinical manifestations and laboratory examination results.

**5. Diagnosis and Classification**	
**5.1 Suspected cases**	Comply with any of the provisions of 3.2.1, 3.2.2, or with the provisions of 3.1
**5.2 Clinically diagnosed cases**	Suspected cases meet the requirements of 3.3.1 at the same time
**5.3 Confirmed cases**	The clinically diagnosed cases also conform to any of the provisions of 3.3.2 and 3.3.3 in laboratory tests.
**Chinese Standard Number**	WS 274-2007
**Issued by**	National Health Commission of the People's Republic of China
**Date issued**	April 17, 2007
**Official Source**	http://www.nhc.gov.cn/ewebeditor/uploadfile/2014/10/20141010173745664.PDF

Supplementary Table 11: The diagnostic criteria for scarlet fever in China

Standard Name	Diagnostic Criteria of Scarlet Fever
**1 Scope**	This standard applies to nationwide medical institutions and their staff to diagnose and report scarlet fever.
**2 Terms and definitions**	2.1 White strawberry tongue<br>The tongue has a white coating on it, while the papillae of the tongue are swollen and reddened, standing out on the white coating, making the tongue resemble a strawberry.<br>2.2 Red strawberry tongue<br>White strawberry tongue following the desquamating process, or the shedding of the tissue which created the white coating) the whiteness disappears while the red and enlarged papillae give it the "red strawberry" appearance.<br>2.3 Pastia's lines<br>Lines of petechiae which appear as pink/red areas located in arm pits and elbow pits.<br>2.4 Circumoral pallor<br>Obvious facial hyperemia compared to hyperemia of the nose and mouth, appearing to be white.
**3 Diagnostic basis**	
**3.1 Epidemiologic Linkage**	Local occurrence and prevalence of the disease, with exposure to scarlet fever patients or to tonsillitis, angina, otitis media, erysipelas or other streptococcal infected patients.

**3.2 Clinical manifestations**	3.2.1 Common scarlet fever<br>Abrupt onset with fever, angina, and rash. Rash is observed on the second day of fever; the skin has disseminated hyperemia and flushing, among which congestive rash as small as a needle's point can be seen that fades with pressing, accompanied by itching. A small number of patients can have a rash with yellow-white pustules, which do not break easily. Pastia's lines may form on skin creases. Facial hyperemia emerges without rash, accompanied by "circumoral pallor". In early onset of the disease, "white strawberry tongue" is seen and is more severe toward the edge of the tongue. After 2-3 days the white coating begins to fall off, forming a "red strawberry tongue". After 2-5 days the rash subsides, after which the skin has desquamation or furfur.<br>3.2.2 Mild scarlet fever<br>Fever, angina and rash are mild and short of duration; desquamation is also mild.<br>3.2.3 Toxic type<br>The main clinical manifestation is toxemia, with obvious poisoning symptoms, like fever, headache, vomiting, hemorrhagic rash, confusion, etc. Angina is not severe. Toxic myocarditis, toxic hepatitis and septic shock can occur.<br>3.2.4 Sepsis type<br>Pharyngeal swelling with exudation of pus or even ulcers, causing cervical lymphadenitis, acute otitis media, acute sinusitis, etc. Can also cause sepsis.<br>3.2.5 Surgical and obstetric type<br>Pathogenic bacteria invade from the wound or birth canal. Local rash at first, which extends to the whole body with no pharyngitis. Systemic symptoms are mostly mild.
**3.3 Laboratory Tests**	3.3.1 The total number of leukocytes and neutrophils increases, with possible toxic granulation for severe patients.<br>3.3.2 Group A *Streptococcus by* rapid antigen detection is positive.<br>3.3.3 The result of bacteria identification is the β hemolytic streptococcus by bacterial culture and by microscopy.<br>3.3.4 The result of a bacitracin-sensitive test is positive.<br>3.3.5 The result of biochemical identification is *Streptococcus pyogenes.*<br>3.3.6 Throat swab or other focal secretion is identified as Group A *β hemolytic streptococcus* by bacteria seroty

**4. Diagnostic principles**	Comprehensive diagnosis should be based on epidemiological data, clinical manifestations and laboratory tests. Confirmation must be based on etiological examination.
**5. Diagnosis and Classification**	
**5.1 Suspected cases**	Satisfying clinical manifestations in 3.2 and 3.3.1.
**5.2 Clinically diagnosed cases**	Meeting any of the following for diagnosis:<br>5.2.1 Probable cases satisfying 3.1.<br>5.2.2 Probable cases satisfying at least one of 3.3.2, 3.3.3, 3.3.4, or 3.3.5.
**5.3 Confirmed cases**	Clinical diagnosis of cases satisfying 3.3.6.
**Chinese Standard Number**	WS282-2008
**Issued by**	National Health Commission of the People's Republic of China
**Date issued**	February 28, 2008
**Official Source**	http://www.nhc.gov.cn/wjw/s9491/200802/38805/files/2c4dd2444eb24922afca4bb3abed613c.pdf

Supplementary Table 12. The age-specific constituent ratio for ten respiratory infectious diseases in China during 2004-2020

Age group	Pertussis	Scarlet Fever	Rubella	TB	pH1N1	Avian H5N1 influenza	Epidemic Cerebrospinal Meningitis	Seasonal influenza	Measles	Mumps	Total RIDs
**0-**	66.6667	9.0909	50.0000	0.1414	2.7293	0.0000	21.4851	2.5316	48.9496	8.0000	1.1622
**1-**	24.2424	0.0000	0.0000	0.0485	2.5109	0.0000	11.6832	2.8129	14.2857	8.0000	0.5178
**2-**	6.0606	0.0000	0.0000	0.0359	1.9651	0.0000	8.1188	3.9381	9.4538	0.0000	0.3795
**3-**	0.0000	9.0909	0.0000	0.0317	2.1834	0.0000	6.1386	2.6723	6.0924	16.0000	0.2965
**4-**	0.0000	18.1818	0.0000	0.0190	1.4192	0.0000	5.3465	3.3755	3.7815	4.0000	0.2392
**5-**	0.0000	0.0000	16.6667	0.0211	0.8734	0.0000	2.0792	1.4065	1.4706	4.0000	0.1146
**6-**	0.0000	27.2727	0.0000	0.0232	0.5459	0.0000	2.7723	1.9691	1.8908	4.0000	0.1403
**7-**	0.0000	0.0000	0.0000	0.0211	0.9825	0.0000	2.0792	1.1252	1.4706	8.0000	0.1127
**8-**	0.0000	0.0000	0.0000	0.0380	0.7642	0.0000	1.2871	1.1252	1.2605	0.0000	0.1028
**9-**	3.0303	0.0000	0.0000	0.0127	0.9825	3.0303	1.4851	0.9845	1.0504	4.0000	0.0889
**10-**	0.0000	18.1818	16.6667	0.2575	2.7293	3.0303	11.1881	2.2504	3.5714	24.0000	0.5989
**15-**	0.0000	9.0909	0.0000	1.3742	5.5677	12.1212	7.7228	1.2658	1.0504	8.0000	1.5832
**20-**	0.0000	0.0000	16.6667	2.3684	13.3188	24.2424	3.5644	2.1097	1.6807	8.0000	2.5971
**25-**	0.0000	0.0000	0.0000	2.6132	10.9170	9.0909	2.5743	3.5162	0.8403	0.0000	2.7592
**30-**	0.0000	0.0000	0.0000	3.0481	6.4410	15.1515	1.3861	3.5162	0.4202	0.0000	3.0616
**35-**	0.0000	0.0000	0.0000	3.8122	7.2052	12.1212	2.3762	4.9226	0.6303	0.0000	3.8304
**40-**	0.0000	0.0000	0.0000	4.8064	8.4061	12.1212	2.2772	7.1730	0.2101	0.0000	4.8088
**45-**	0.0000	9.0909	0.0000	5.9062	5.7860	3.0303	1.1881	6.8917	0.6303	0.0000	5.7654
**50-**	0.0000	0.0000	0.0000	7.0651	4.2576	3.0303	1.7822	5.6259	0.4202	0.0000	6.8129

**55-**	0.0000	0.0000	0.0000	7.7405	6.7686	0.0000	1.1881	5.2039	0.2101	0.0000	7.4691
**60-**	0.0000	0.0000	0.0000	9.5517	3.7118	3.0303	0.4950	7.1730	0.4202	0.0000	9.1274
**65-**	0.0000	0.0000	0.0000	10.8519	3.0568	0.0000	0.8911	5.6259	0.2101	0.0000	10.3152
**70-**	0.0000	0.0000	0.0000	13.6235	2.9476	0.0000	0.3960	5.4852	0.0000	4.0000	12.8965
**75-**	0.0000	0.0000	0.0000	12.7222	2.5109	0.0000	0.4950	5.3446	0.0000	0.0000	12.0427
**80-**	0.0000	0.0000	0.0000	9.0007	1.0917	0.0000	0.0000	5.6259	0.0000	0.0000	8.5265
**≥85**	0.0000	0.0000	0.0000	4.8655	0.3275	0.0000	0.0000	6.3291	0.0000	0.0000	4.6507

Supplementary Table 13. The age-specific case fatality rate (‰) for ten respiratory infectious diseases in China during 2004-2020

Age Groups	Pertussis	TB	Rubella	Influenza	Epidemic Cerebrospinal Meningitis	Mumps	Measles	H5N1	pH1N1	Scarlet fever	Total RIDs
0-	0.3709	5.8829	0.1173	0.0584	242.1875	0.1286	1.0918	0	14.1643	0	0.9161
1-	0.515	3.1297	0	0.0436	217.3112	0.0402	0.918	0	11.552	0	0.4121
2-	0.2706	2.2336	0	0.0592	171.9078	0	0.893	0	7.0922	0	0.2763
3-	0	2.0254	0	0.035	164.0212	0.0166	0.8094	0	5.94	0	0.1705
4-	0	1.1235	0	0.0495	146.7391	0.0029	0.5869	0	3.9562	0.0276	0.1715
5-	0	1.2115	0.048	0.0229	61.7647	0.0024	0.259	0	2.1192	0.0114	0.0552
6-	0	1.1763	0	0.0309	80.2292	0.0023	0.3568	0	1.0858	0.0232	0.1737
7-	0	1.0298	0	0.0185	57.5342	0.0046	0.3135	0	1.6023	0	0.0555
8-	0	1.7859	0	0.0254	37.9009	0	0.3075	0	1.1377	0	0.0614
9-	1.0235	0.602	0	0.0289	44.1176	0.0028	0.3387	500	1.3139	0	0.0647
10-	0	1.0846	0.007	0.0198	52.6561	0.0052	0.3682	1000	0.5969	0.0992	0.1311
15-	0	0.6792	0	0.0257	43.9932	0.0068	0.1409	1000	1.1295	0	0.4408
20-	0	0.7123	0.0187	0.0642	52.3256	0.021	0.1665	888.889	6.9192	0	0.648
25-	0	0.95	0	0.0761	74.2857	0	0.0738	600	9.8107	0	0.7712
30-	0	1.2946	0	0.0821	42.4242	0	0.0443	555.556	12.2661	0	0.9904
35-	0	1.6157	0	0.1668	70.1754	0	0.0989	1000	17.3047	0	1.3591
40-	0	1.8626	0	0.3374	77.1812	0	0.0603	800	26.8386	0	1.7012
45-	0	2.2746	0	0.3172	50	0	0.3991	1000	23.0135	0	2.054

50-	0	2.5007	0	0.2578	81.448	0	0.6412	500	17.1353	0	2.2698
55-	0	2.8152	0	0.2688	84.507	0	0.993	0	31.031	0	2.5916
60-	0	3.3467	0	0.3656	56.1798	0	5.3476	1000	30.881	0	3.07
65-	0	4.2637	0	0.3527	136.3636	0	5.4945	0	47.2175	0	3.9326
70-	0	5.9801	0	0.5047	102.5641	0.244	0	0	48.913	0	5.6165
75-	0	8.1218	0	0.6938	238.0952	0	0	0	59.126	0	7.6161
80-	0	11.431	0	1.0044	0	0	0	0	62.1118	0	10.4082
≥ 85	0	15.984	0	1.6519	0	0	0	0	49.1803	0	13.6551

Supplementary Table 14. Provincial-level mortality from mumps in China, 2004-2020

Area	2004	2005	2006	2007	2008	2009	2010	2011	2012	2013	2014	2015	2016	2017	2018	2019	2020	Mean mortality (per 100 000)	Overall trend
Beijing	0.0000	0.0000	0.0000	0.0000	0.0000	0.0000	0.0000	0.0000	0.0000	0.0000	0.0000	0.0000	0.0000	0.0000	0.0000	0.0000	0.0000	0.0000	0.0000
Tianjin	0.0000	0.0000	0.0000	0.0000	0.0000	0.0000	0.0000	0.0000	0.0000	0.0000	0.0000	0.0000	0.0000	0.0000	0.0000	0.0000	0.0000	0.0000	0.0000
Hebei	0.0000	0.0000	0.0000	0.0000	0.0000	0.0000	0.0014	0.0000	0.0000	0.0000	0.0000	0.0000	0.0000	0.0000	0.0000	0.0000	0.0000	0.0001	0.0000
Shanxi	0.0000	0.0000	0.0000	0.0000	0.0000	0.0000	0.0000	0.0000	0.0000	0.0000	0.0000	0.0000	0.0000	0.0000	0.0000	0.0000	0.0000	0.0000	0.0000
Inner Mongolia	0.0000	0.0000	0.0000	0.0000	0.0000	0.0000	0.0000	0.0000	0.0000	0.0000	0.0040	0.0000	0.0000	0.0000	0.0000	0.0000	0.0000	0.0002	0.0000
Liaoning	0.0000	0.0000	0.0000	0.0000	0.0000	0.0000	0.0000	0.0000	0.0000	0.0000	0.0000	0.0000	0.0000	0.0000	0.0000	0.0000	0.0000	0.0000	0.0000
Jilin	0.0000	0.0000	0.0000	0.0000	0.0000	0.0000	0.0000	0.0000	0.0000	0.0000	0.0000	0.0000	0.0000	0.0000	0.0000	0.0000	0.0000	0.0000	0.0000
Heilongjiang	0.0000	0.0000	0.0000	0.0000	0.0000	0.0000	0.0026	0.0000	0.0000	0.0000	0.0000	0.0000	0.0000	0.0000	0.0000	0.0000	0.0000	0.0002	0.0000
Shanghai	0.0000	0.0000	0.0000	0.0000	0.0000	0.0053	0.0000	0.0000	0.0000	0.0000	0.0000	0.0000	0.0000	0.0000	0.0000	0.0000	0.0000	0.0003	0.0000
Jiangsu	0.0000	0.0000	0.0000	0.0000	0.0000	0.0000	0.0000	0.0000	0.0000	0.0013	0.0000	0.0000	0.0000	0.0000	0.0000	0.0000	0.0000	0.0001	0.0000
Zhejiang	0.0000	0.0000	0.0000	0.0020	0.0000	0.0000	0.0000	0.0000	0.0000	0.0000	0.0000	0.0000	0.0000	0.0000	0.0000	0.0000	0.0000	0.0001	0.0000
Anhui	0.0000	0.0000	0.0000	0.0000	0.0000	0.0000	0.0016	0.0000	0.0000	0.0000	0.0000	0.0000	0.0000	0.0000	0.0000	0.0000	0.0000	0.0001	0.0000
Fujian	0.0000	0.0000	0.0000	0.0000	0.0000	0.0000	0.0000	0.0000	0.0000	0.0000	0.0000	0.0000	0.0000	0.0000	0.0000	0.0000	0.0000	0.0000	0.0000
Jiangxi	0.0000	0.0000	0.0046	0.0000	0.0000	0.0000	0.0000	0.0000	0.0000	0.0000	0.0000	0.0000	0.0000	0.0000	0.0000	0.0000	0.0000	0.0003	0.0000
Shandong	0.0000	0.0000	0.0000	0.0000	0.0011	0.0000	0.0000	0.0000	0.0000	0.0000	0.0000	0.0000	0.0000	0.0000	0.0000	0.0000	0.0000	0.0001	0.0000
Henan	0.0000	0.0000	0.0011	0.0000	0.0000	0.0000	0.0000	0.0011	0.0000	0.0000	0.0000	0.0000	0.0000	0.0000	0.0000	0.0000	0.0010	0.0002	0.0010
Hubei	0.0000	0.0000	0.0000	0.0000	0.0000	0.0000	0.0000	0.0000	0.0000	0.0000	0.0000	0.0000	0.0000	0.0000	0.0000	0.0000	0.0000	0.0000	0.0000
Hunan	0.0000	0.0000	0.0000	0.0000	0.0000	0.0000	0.0000	0.0000	0.0000	0.0000	0.0000	0.0000	0.0000	0.0000	0.0000	0.0000	0.0000	0.0000	0.0000
Guangdong	0.0000	0.0011	0.0011	0.0000	0.0000	0.0000	0.0000	0.0000	0.0000	0.0000	0.0000	0.0000	0.0000	0.0000	0.0000	0.0000	0.0000	0.0001	0.0000
Guangxi	0.0021	0.0000	0.0000	0.0000	0.0021	0.0000	0.0000	0.0000	0.0000	0.0000	0.0000	0.0000	0.0000	0.0021	0.0000	0.0000	0.0000	0.0004	-0.0021
Hainan	0.0000	0.0000	0.0000	0.0000	0.0000	0.0000	0.0000	0.0000	0.0000	0.0000	0.0000	0.0000	0.0000	0.0000	0.0000	0.0000	0.0000	0.0000	0.0000
Chongqing	0.0000	0.0036	0.0000	0.0000	0.0000	0.0000	0.0000	0.0000	0.0000	0.0000	0.0000	0.0000	0.0000	0.0000	0.0000	0.0000	0.0000	0.0002	0.0000
Sichuan	0.0000	0.0000	0.0000	0.0000	0.0000	0.0000	0.0000	0.0000	0.0000	0.0000	0.0000	0.0000	0.0000	0.0000	0.0000	0.0000	0.0000	0.0000	0.0000
Guizhou	0.0000	0.0000	0.0000	0.0000	0.0000	0.0000	0.0026	0.0000	0.0000	0.0000	0.0000	0.0000	0.0000	0.0000	0.0000	0.0000	0.0000	0.0002	0.0000
Yunnan	0.0000	0.0000	0.0000	0.0000	0.0000	0.0000	0.0000	0.0000	0.0000	0.0000	0.0000	0.0000	0.0000	0.0000	0.0000	0.0000	0.0000	0.0000	0.0000
Tibet	0.0000	0.0000	0.0000	0.0000	0.0000	0.0000	0.0000	0.0000	0.0000	0.0000	0.0000	0.0000	0.0000	0.0000	0.0000	0.0000	0.0000	0.0000	0.0000
Shaanxi	0.0082	0.0054	0.0000	0.0000	0.0000	0.0000	0.0000	0.0000	0.0000	0.0000	0.0000	0.0000	0.0000	0.0000	0.0000	0.0000	0.0000	0.0008	-0.0082
Gansu	0.0000	0.0000	0.0000	0.0000	0.0000	0.0000	0.0000	0.0000	0.0000	0.0000	0.0000	0.0000	0.0000	0.0000	0.0000	0.0000	0.0000	0.0000	0.0000
Qinghai	0.0000	0.0000	0.0000	0.0000	0.0000	0.0000	0.0000	0.0000	0.0000	0.0000	0.0000	0.0000	0.0000	0.0000	0.0000	0.0000	0.0000	0.0000	0.0000
Ningxia	0.0000	0.0170	0.0000	0.0000	0.0000	0.0000	0.0000	0.0000	0.0000	0.0000	0.0000	0.0000	0.0000	0.0000	0.0000	0.0000	0.0000	0.0010	0.0000
Xinjiang	0.0000	0.0000	0.0000	0.0000	0.0000	0.0000	0.0000	0.0000	0.0000	0.0000	0.0000	0.0000	0.0000	0.0000	0.0000	0.0000	0.0000	0.0000	0.0000

Notes: Overall trend=the mortality in 2020- the mortality in 2004.

Supplementary Table 15. Provincial-level mortality from rubella in China, 2004-2020

Area	2004	2005	2006	2007	2008	2009	2010	2011	2012	2013	2014	2015	2016	2017	2018	2019	2020	Mean mortality (per 100 000)	Overall trend
Beijing	0.0000	0.0000	0.0000	0.0000	0.0000	0.0000	0.0000	0.0000	0.0000	0.0000	0.0000	0.0000	0.0000	0.0000	0.0000	0.0000	0.0000	0.0000	0.0000
Tianjin	0.0000	0.0000	0.0000	0.0000	0.0000	0.0000	0.0000	0.0000	0.0000	0.0000	0.0000	0.0000	0.0000	0.0000	0.0000	0.0000	0.0000	0.0000	0.0000
Hebei	0.0000	0.0000	0.0000	0.0000	0.0000	0.0000	0.0014	0.0000	0.0000	0.0000	0.0000	0.0014	0.0000	0.0000	0.0000	0.0000	0.0000	0.0002	0.0000
Shanxi	0.0000	0.0000	0.0000	0.0000	0.0000	0.0000	0.0000	0.0000	0.0000	0.0000	0.0000	0.0000	0.0000	0.0000	0.0000	0.0000	0.0000	0.0000	0.0000
Inner Mongolia	0.0000	0.0000	0.0000	0.0000	0.0000	0.0000	0.0000	0.0000	0.0000	0.0000	0.0000	0.0000	0.0000	0.0000	0.0000	0.0000	0.0000	0.0000	0.0000
Liaoning	0.0000	0.0000	0.0000	0.0000	0.0000	0.0000	0.0000	0.0000	0.0000	0.0000	0.0000	0.0000	0.0000	0.0000	0.0000	0.0000	0.0000	0.0000	0.0000
Jilin	0.0000	0.0000	0.0000	0.0000	0.0000	0.0000	0.0000	0.0000	0.0000	0.0000	0.0000	0.0000	0.0000	0.0000	0.0000	0.0000	0.0000	0.0000	0.0000
Heilongjiang	0.0000	0.0000	0.0000	0.0000	0.0000	0.0000	0.0000	0.0000	0.0000	0.0000	0.0000	0.0000	0.0000	0.0000	0.0000	0.0000	0.0000	0.0000	0.0000
Shanghai	0.0000	0.0000	0.0000	0.0000	0.0000	0.0000	0.0000	0.0000	0.0000	0.0000	0.0000	0.0000	0.0000	0.0000	0.0000	0.0000	0.0000	0.0000	0.0000
Jiangsu	0.0000	0.0000	0.0000	0.0000	0.0000	0.0000	0.0000	0.0000	0.0000	0.0000	0.0000	0.0000	0.0000	0.0000	0.0000	0.0000	0.0000	0.0000	0.0000
Zhejiang	0.0000	0.0000	0.0000	0.0000	0.0000	0.0000	0.0000	0.0000	0.0000	0.0000	0.0000	0.0000	0.0000	0.0000	0.0000	0.0000	0.0000	0.0000	0.0000
Anhui	0.0000	0.0000	0.0000	0.0000	0.0000	0.0000	0.0000	0.0000	0.0000	0.0000	0.0000	0.0000	0.0000	0.0000	0.0000	0.0000	0.0000	0.0000	0.0000
Fujian	0.0000	0.0000	0.0000	0.0000	0.0000	0.0000	0.0000	0.0000	0.0000	0.0000	0.0000	0.0000	0.0000	0.0000	0.0000	0.0000	0.0000	0.0000	0.0000
Jiangxi	0.0000	0.0000	0.0000	0.0000	0.0000	0.0000	0.0000	0.0000	0.0000	0.0000	0.0000	0.0000	0.0000	0.0000	0.0000	0.0000	0.0000	0.0000	0.0000
Shandong	0.0000	0.0000	0.0000	0.0000	0.0000	0.0000	0.0000	0.0000	0.0000	0.0000	0.0000	0.0000	0.0000	0.0000	0.0000	0.0000	0.0000	0.0000	0.0000
Henan	0.0010	0.0000	0.0000	0.0000	0.0000	0.0000	0.0000	0.0000	0.0000	0.0000	0.0000	0.0000	0.0000	0.0000	0.0000	0.0000	0.0000	0.0001	-0.0010
Hubei	0.0000	0.0000	0.0000	0.0000	0.0000	0.0000	0.0000	0.0000	0.0000	0.0000	0.0000	0.0000	0.0000	0.0000	0.0000	0.0000	0.0000	0.0000	0.0000
Hunan	0.0000	0.0000	0.0000	0.0000	0.0000	0.0000	0.0000	0.0000	0.0000	0.0000	0.0000	0.0000	0.0000	0.0000	0.0000	0.0000	0.0000	0.0000	0.0000
Guangdong	0.0000	0.0000	0.0000	0.0000	0.0000	0.0000	0.0000	0.0000	0.0010	0.0000	0.0000	0.0000	0.0000	0.0000	0.0000	0.0000	0.0000	0.0001	0.0000
Guangxi	0.0000	0.0000	0.0000	0.0000	0.0000	0.0000	0.0000	0.0000	0.0000	0.0000	0.0000	0.0000	0.0000	0.0000	0.0000	0.0000	0.0000	0.0000	0.0000
Hainan	0.0000	0.0000	0.0000	0.0000	0.0000	0.0000	0.0000	0.0000	0.0000	0.0000	0.0000	0.0000	0.0000	0.0000	0.0000	0.0000	0.0000	0.0000	0.0000
Chongqing	0.0000	0.0000	0.0000	0.0000	0.0000	0.0000	0.0000	0.0000	0.0000	0.0000	0.0000	0.0000	0.0000	0.0000	0.0000	0.0000	0.0000	0.0000	0.0000
Sichuan	0.0000	0.0000	0.0000	0.0000	0.0000	0.0000	0.0000	0.0000	0.0000	0.0000	0.0000	0.0000	0.0000	0.0000	0.0000	0.0000	0.0000	0.0000	0.0000
Guizhou	0.0000	0.0000	0.0000	0.0000	0.0000	0.0000	0.0000	0.0000	0.0000	0.0000	0.0000	0.0000	0.0000	0.0000	0.0000	0.0000	0.0000	0.0000	0.0000
Yunnan	0.0000	0.0000	0.0000	0.0000	0.0000	0.0000	0.0000	0.0000	0.0000	0.0000	0.0000	0.0000	0.0000	0.0000	0.0000	0.0000	0.0000	0.0000	0.0000
Tibet	0.0000	0.0000	0.0000	0.0000	0.0000	0.0000	0.0000	0.0000	0.0000	0.0000	0.0000	0.0000	0.0000	0.0000	0.0000	0.0000	0.0000	0.0000	0.0000
Shaanxi	0.0000	0.0000	0.0000	0.0000	0.0000	0.0000	0.0000	0.0000	0.0000	0.0000	0.0000	0.0000	0.0000	0.0000	0.0000	0.0000	0.0000	0.0000	0.0000
Gansu	0.0000	0.0000	0.0000	0.0000	0.0000	0.0000	0.0000	0.0000	0.0000	0.0000	0.0000	0.0000	0.0000	0.0000	0.0000	0.0000	0.0000	0.0000	0.0000
Qinghai	0.0000	0.0000	0.0000	0.0000	0.0000	0.0000	0.0000	0.0000	0.0000	0.0000	0.0000	0.0000	0.0000	0.0000	0.0000	0.0000	0.0000	0.0000	0.0000
Ningxia	0.0000	0.0170	0.0000	0.0000	0.0000	0.0000	0.0000	0.0000	0.0000	0.0000	0.0000	0.0000	0.0000	0.0000	0.0000	0.0000	0.0000	0.0010	0.0000
Xinjiang	0.0000	0.0000	0.0000	0.0000	0.0048	0.0000	0.0000	0.0000	0.0000	0.0000	0.0000	0.0000	0.0000	0.0000	0.0000	0.0000	0.0000	0.0003	0.0000

Notes: Overall trend=the mortality in 2020- the mortality in 2004.

Supplementary Table 16. Provincial-level mortality from pertussis in China, 2004-2020

Area	2004	2005	2006	2007	2008	2009	2010	2011	2012	2013	2014	2015	2016	2017	2018	2019	2020	Mean mortality (per 100 000)	Overall trend
Beijing											0.0047	0.0047	0.0139		0.0000	0.0000	0.0000	0.0039	0.0000
Tianjin							0.0081						0.0000		0.0000	0.0000	0.0000	0.0016	0.0000
Hebei													0.0000		0.0000	0.0000	0.0000	0.0000	0.0000
Shanxi													0.0000		0.0000	0.0000	0.0000	0.0000	0.0000
Inner Mongolia													0.0000		0.0000	0.0000	0.0000	0.0000	0.0000
Liaoning													0.0000		0.0000	0.0000	0.0000	0.0000	0.0000
Jilin						0.0037							0.0000		0.0000	0.0000	0.0000	0.0007	0.0000
Heilongjiang													0.0000		0.0000	0.0000	0.0000	0.0000	0.0000
Shanghai													0.0000		0.0000	0.0000	0.0000	0.0000	0.0000
Jiangsu			0.0027										0.0000		0.0000	0.0000	0.0000	0.0005	0.0000
Zhejiang													0.0000		0.0000	0.0000	0.0000	0.0000	0.0000
Anhui													0.0000		0.0000	0.0000	0.0000	0.0000	0.0000
Fujian													0.0000		0.0000	0.0000	0.0000	0.0000	0.0000
Jiangxi			0.0023										0.0000		0.0000	0.0000	0.0000	0.0005	0.0000
Shandong		0.0011						0.0010			0.0010		0.0000		0.0010	0.0000	0.0000	0.0006	0.0000
Henan													0.0000		0.0000	0.0000	0.0000	0.0000	0.0000
Hubei													0.0000		0.0000	0.0000	0.0000	0.0000	0.0000
Hunan													0.0000		0.0000	0.0000	0.0000	0.0000	0.0000
Guangdong		0.0011											0.0000		0.0000	0.0009	0.0000	0.0004	0.0000
Guangxi													0.0000		0.0000	0.0020	0.0000	0.0005	0.0000
Hainan													0.0000		0.0000	0.0000	0.0000	0.0000	0.0000
Chongqing	0.0032												0.0000		0.0000	0.0000	0.0000	0.0006	-0.0032
Sichuan	0.0023				0.0012				0.0012				0.0000		0.0012	0.0000	0.0000	0.0009	-0.0023
Guizhou												0.0029	0.0000		0.0000	0.0000	0.0000	0.0006	0.0000
Yunnan													0.0000		0.0000	0.0000	0.0000	0.0000	0.0000
Tibet													0.0000		0.0000	0.0000	0.0000	0.0000	0.0000
Shaanxi													0.0000		0.0000	0.0000	0.0026	0.0006	0.0026
Gansu													0.0000		0.0000	0.0000	0.0000	0.0000	0.0000
Qinghai	0.0556												0.0000		0.0000	0.0000	0.0000	0.0111	-0.0556
Ningxia													0.0000		0.0000	0.0000	0.0000	0.0000	0.0000
Xinjiang	0.0161		0.0050					0.0046					0.0000		0.0000	0.0000	0.0000	0.0037	-0.0161

Notes: Overall trend=the mortality in 2020- the mortality in 2004.

Supplementary Table 17. Provincial-level mortality from scarlet fever in China, 2004-2020

Area	2004	2005	2006	2007	2008	2009	2010	2011	2012	2013	2014	2015	2016	2017	2018	2019	2020	Mean mortality (per 100 000)	Overall trend
Beijing	0.0000	0.0000	0.0000	0.0000	0.0000	0.0000	0.0000	0.0000	0.0000	0.0000	0.0000	0.0000	0.0000	0.0000	0.0000	0.0000	0.0000	0.0000	0.0000
Tianjin	0.0000	0.0000	0.0000	0.0000	0.0000	0.0000	0.0000	0.0000	0.0000	0.0000	0.0000	0.0000	0.0000	0.0000	0.0000	0.0000	0.0000	0.0000	0.0000
Hebei	0.0000	0.0000	0.0000	0.0000	0.0000	0.0000	0.0000	0.0000	0.0000	0.0000	0.0000	0.0000	0.0000	0.0000	0.0000	0.0000	0.0000	0.0000	0.0000
Shanxi	0.0000	0.0000	0.0000	0.0000	0.0000	0.0000	0.0000	0.0000	0.0000	0.0000	0.0000	0.0000	0.0000	0.0000	0.0000	0.0000	0.0000	0.0000	0.0000
Inner Mongolia	0.0000	0.0000	0.0000	0.0000	0.0000	0.0000	0.0000	0.0000	0.0000	0.0000	0.0000	0.0000	0.0000	0.0000	0.0000	0.0000	0.0000	0.0000	0.0000
Liaoning	0.0000	0.0000	0.0000	0.0000	0.0000	0.0000	0.0000	0.0000	0.0000	0.0000	0.0000	0.0023	0.0000	0.0000	0.0000	0.0000	0.0000	0.0001	0.0000
Jilin	0.0000	0.0000	0.0000	0.0000	0.0000	0.0000	0.0000	0.0000	0.0000	0.0000	0.0000	0.0000	0.0000	0.0000	0.0000	0.0000	0.0000	0.0000	0.0000
Heilongjiang	0.0000	0.0026	0.0000	0.0000	0.0000	0.0000	0.0000	0.0026	0.0026	0.0026	0.0000	0.0000	0.0000	0.0000	0.0000	0.0000	0.0000	0.0006	0.0000
Shanghai	0.0000	0.0000	0.0000	0.0000	0.0000	0.0000	0.0000	0.0000	0.0000	0.0000	0.0000	0.0000	0.0000	0.0000	0.0000	0.0000	0.0000	0.0000	0.0000
Jiangsu	0.0000	0.0000	0.0000	0.0000	0.0000	0.0000	0.0000	0.0000	0.0000	0.0013	0.0000	0.0000	0.0000	0.0000	0.0000	0.0000	0.0000	0.0001	0.0000
Zhejiang	0.0000	0.0000	0.0000	0.0000	0.0000	0.0000	0.0000	0.0000	0.0000	0.0000	0.0000	0.0000	0.0000	0.0000	0.0000	0.0000	0.0000	0.0000	0.0000
Anhui	0.0000	0.0000	0.0000	0.0000	0.0000	0.0000	0.0000	0.0000	0.0000	0.0000	0.0000	0.0000	0.0000	0.0000	0.0000	0.0000	0.0000	0.0000	0.0000
Fujian	0.0000	0.0000	0.0000	0.0000	0.0000	0.0000	0.0000	0.0000	0.0000	0.0000	0.0000	0.0000	0.0000	0.0000	0.0000	0.0000	0.0000	0.0000	0.0000
Jiangxi	0.0000	0.0000	0.0000	0.0000	0.0000	0.0000	0.0000	0.0000	0.0000	0.0000	0.0000	0.0000	0.0000	0.0000	0.0000	0.0000	0.0000	0.0000	0.0000
Shandong	0.0000	0.0000	0.0000	0.0011	0.0000	0.0000	0.0000	0.0000	0.0000	0.0000	0.0000	0.0000	0.0000	0.0000	0.0000	0.0000	0.0000	0.0001	0.0000
Henan	0.0000	0.0000	0.0000	0.0000	0.0000	0.0000	0.0000	0.0000	0.0000	0.0000	0.0000	0.0000	0.0000	0.0000	0.0000	0.0000	0.0000	0.0000	0.0000
Hubei	0.0000	0.0000	0.0000	0.0000	0.0000	0.0000	0.0000	0.0000	0.0000	0.0000	0.0000	0.0000	0.0000	0.0000	0.0000	0.0000	0.0000	0.0000	0.0000
Hunan	0.0000	0.0000	0.0000	0.0000	0.0000	0.0000	0.0000	0.0000	0.0015	0.0000	0.0000	0.0000	0.0000	0.0000	0.0000	0.0000	0.0000	0.0001	0.0000
Guangdong	0.0000	0.0000	0.0000	0.0000	0.0000	0.0000	0.0000	0.0000	0.0000	0.0000	0.0000	0.0000	0.0000	0.0000	0.0000	0.0000	0.0009	0.0001	0.0009
Guangxi	0.0000	0.0000	0.0000	0.0000	0.0000	0.0000	0.0000	0.0000	0.0000	0.0000	0.0000	0.0000	0.0000	0.0000	0.0000	0.0000	0.0000	0.0000	0.0000
Hainan	0.0000	0.0000	0.0000	0.0000	0.0000	0.0000	0.0000	0.0000	0.0000	0.0000	0.0000	0.0000	0.0000	0.0000	0.0000	0.0000	0.0000	0.0000	0.0000
Chongqing	0.0000	0.0000	0.0000	0.0000	0.0000	0.0000	0.0000	0.0000	0.0000	0.0000	0.0000	0.0000	0.0000	0.0000	0.0000	0.0000	0.0000	0.0000	0.0000
Sichuan	0.0000	0.0000	0.0000	0.0000	0.0000	0.0000	0.0000	0.0000	0.0000	0.0000	0.0000	0.0000	0.0000	0.0000	0.0000	0.0000	0.0000	0.0000	0.0000
Guizhou	0.0000	0.0000	0.0000	0.0000	0.0000	0.0000	0.0000	0.0000	0.0000	0.0000	0.0000	0.0000	0.0000	0.0000	0.0000	0.0000	0.0000	0.0000	0.0000
Yunnan	0.0000	0.0023	0.0000	0.0000	0.0000	0.0000	0.0000	0.0000	0.0000	0.0000	0.0000	0.0000	0.0000	0.0000	0.0000	0.0000	0.0000	0.0001	0.0000
Tibet	0.0000	0.0000	0.0000	0.0000	0.0000	0.0000	0.0000	0.0000	0.0000	0.0000	0.0000	0.0000	0.0000	0.0000	0.0000	0.0000	0.0000	0.0000	0.0000
Shaanxi	0.0000	0.0000	0.0000	0.0000	0.0000	0.0000	0.0000	0.0000	0.0000	0.0000	0.0000	0.0000	0.0000	0.0000	0.0000	0.0000	0.0000	0.0000	0.0000
Gansu	0.0000	0.0000	0.0000	0.0000	0.0000	0.0000	0.0000	0.0000	0.0000	0.0000	0.0000	0.0000	0.0000	0.0000	0.0000	0.0000	0.0000	0.0000	0.0000
Qinghai	0.0000	0.0000	0.0000	0.0000	0.0000	0.0000	0.0000	0.0000	0.0000	0.0000	0.0000	0.0000	0.0000	0.0000	0.0000	0.0000	0.0000	0.0000	0.0000
Ningxia	0.0185	0.0000	0.0000	0.0000	0.0000	0.0000	0.0000	0.0000	0.0000	0.0000	0.0000	0.0000	0.0000	0.0000	0.0000	0.0000	0.0000	0.0011	-0.0185
Xinjiang	0.0000	0.0000	0.0000	0.0000	0.0000	0.0000	0.0000	0.0000	0.0000	0.0000	0.0000	0.0000	0.0000	0.0000	0.0000	0.0000	0.0000	0.0000	0.0000

Notes: Overall trend=the mortality in 2020- the mortality in 2004.
